# Cell reorientation on a cyclically strained substrate

**DOI:** 10.1093/pnasnexus/pgac199

**Published:** 2022-09-22

**Authors:** Shuvrangsu Das, Alberto Ippolito, Patrick McGarry, Vikram S Deshpande

**Affiliations:** Department of Engineering, Cambridge University, Trumpington St, Cambridge CB2 1PZ, UK; Department of Engineering, Cambridge University, Trumpington St, Cambridge CB2 1PZ, UK; Department of Mechanical and Biomedical Engineering, National University of Ireland, University Road, Galway H91 CF50, Ireland; Department of Engineering, Cambridge University, Trumpington St, Cambridge CB2 1PZ, UK

**Keywords:** cyclic strain avoidance, stress-fiber alignment, homeostasis, fluctuations

## Abstract

Cyclic strain avoidance, the phenomenon of cell and cytoskeleton alignment perpendicular to the direction of cyclic strain of the underlying 2D substrate, is an important characteristic of the adherent cell organization. This alignment has typically been attributed to the stress-fiber reorganization although observations clearly show that stress-fiber reorganization under cyclic loading is closely coupled to cell morphology and reorientation of the cells. Here, we develop a statistical mechanics framework that couples the cytoskeletal stress-fiber organization with cell morphology under imposed cyclic straining and make quantitative comparisons with observations. The framework accurately predicts that cyclic strain avoidance stems primarily from cell reorientation away from the cyclic straining rather than cytoskeletal reorganization within the cell. The reorientation of the cell is a consequence of the cell lowering its free energy by largely avoiding the imposed cyclic straining. Furthermore, we investigate the kinetics of the cyclic strain avoidance mechanism and demonstrate that it emerges primarily due to the rigid body rotation of the cell rather than via a trajectory involving cell straining. Our results provide clear physical insights into the coupled dynamics of cell morphology and stress-fibers, which ultimately leads to cellular organization in cyclically strained tissues.

Significance Statement Cellular organization dictates the biological and mechanical properties of tissues in part because cells exert forces on their surrounding primarily in the direction they are aligned. This orientational arrangement of cells in tissues is strongly influenced by cyclic straining that is often experienced in vivo. We have developed a statistical mechanics framework that couples the mechano-chemistry of the stress-fiber cytoskeleton with cell morphology under imposed cyclic loading. The model shows that the cyclic strain avoidance (the tendency of cells to reorient away from the cyclic straining direction) emerges as a consequence of cells attempting to lower their free-energies. Our numerical framework is expected to form an essential component to help design functional tissue engineered systems.

## Introduction

Mechanical interactions of cells with their environment are known to strongly influence the morphological and biochemical responses of cells. For example, it is well known that a reduced mechanical stiffness of the substrate leads to a decrease in cell spreading (1, 2), elongation (3,4), and cytoskeletal ordering (3, 5). Similarly, cells seeded on substrates with ligand patterns or heterogeneous elasticity respond by forming actin and focal adhesion distributions that typically align with the heterogeneity (6–8). This mechanosensitivity of adherent cells is mediated by a series of protein complexes, including the transmembrane focal adhesions and the dynamic network of intracellular proteins, such as stress-fibers (SFs).

**Fig. 1.**
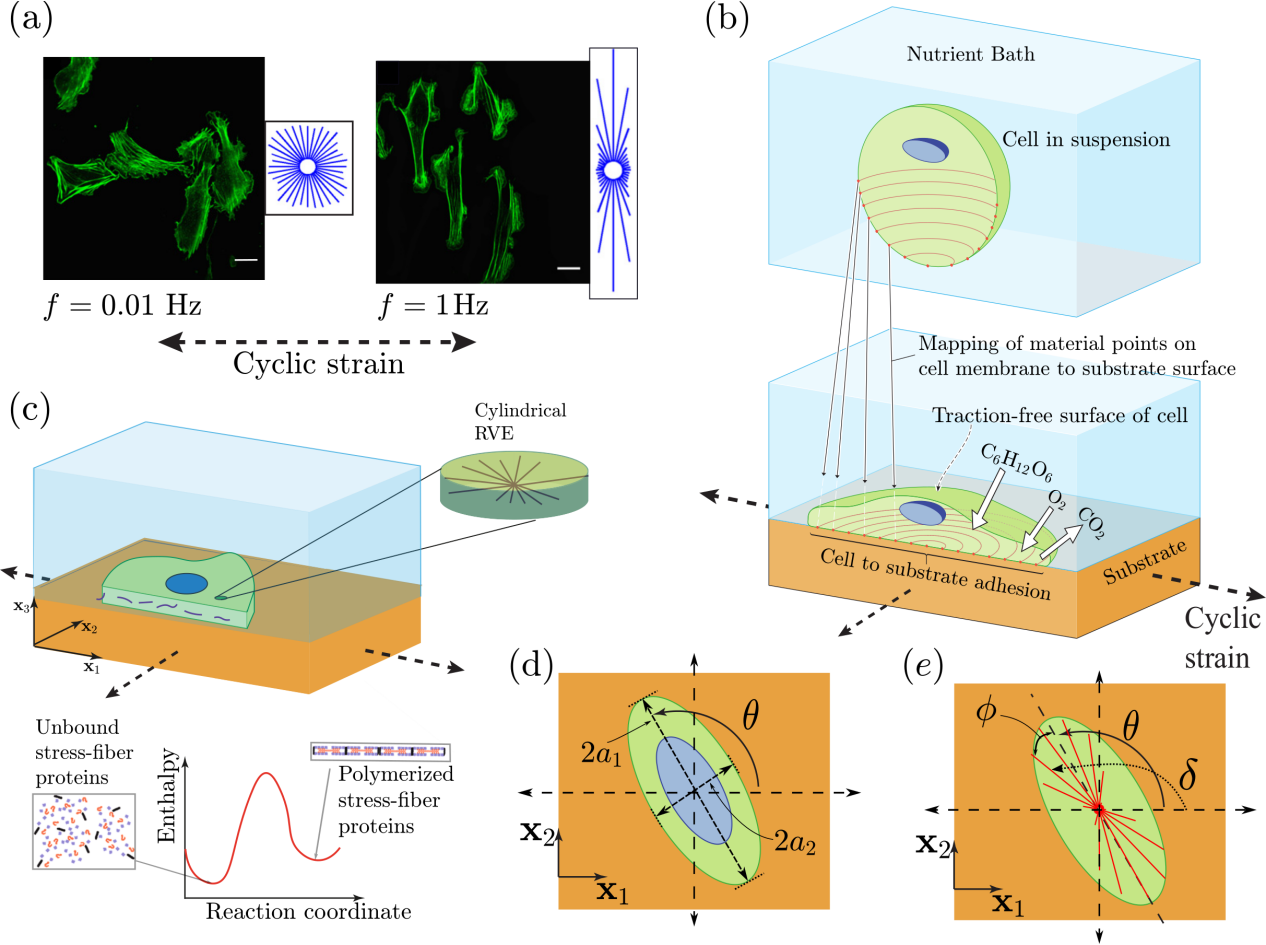
(a) Immunofluorescence images showing actin distributions within U2OS cells subjected to a uniaxial cyclic strain with a stretch amplitude $10\%$ at frequencies *f* = 0.01 and 1 Hz for around 12 hours. Reproduced from (14). Scale bar 25 μm. (b) Sketch showing a single cell adhered to a substrate subjected to a biaxial cyclic strain in the ${\mathbf{ x}}_1\hbox{--}{\mathbf{ x}}_2$ plane. The cell exchanges high energy nutrients with the nutrient bath. A morphological microstate is defined by the mapping of material points on the cell membrane with material points on the substrate. (c) The 2D approximation of the cells. The components of the cell that are modeled explicitly include an elastic nucleus and cytoplasm as well as the contractile SFs in their polymerized state along with the the unbound components that are free to diffuse within the cytoplasm. (d) The elliptical approximation of the cell as a spatially uniform ellipse on the cyclically loaded substrate. The principal axes 2*a*_1_ and 2*a*_2_, respectively, of the ellipse are labeled along with the definition of the orientation *θ* of the cell. (e) Sketches to illustrate the orientation *δ* of an SF relative to the x_1_ imposed cyclic strain direction and the orientation *ϕ* of the SF relative to the major axis of the ellipse.

Mechanosensitivity also affects the behavior of cells when they are subjected to external forces and/or deformations. For example, there exists a wealth of in vitro data to simulate the cyclic strain experienced by endothelial cells (9). In these experiments, cells are seeded on 2D substrates that are stiffer than the cells and the substrates subjected to uniaxial cyclic straining; cells orient away from the imposed cyclic strain direction and this behavior is widely known as cellular strain avoidance (10–13). Typically, the phenomenon of strain avoidance increases with increasing loading frequency (Fig. 1a) and strain amplitude (12,14). Alignment behavior has also been shown to be reported in 3D tissues where cells are seeded in a collagen matrix, although now the precise boundary conditions on the 3D tissue play a more crucial role (15–17).

Under cyclic loading, cells assume a diversity of shapes but intriguingly with increasing frequency and amplitude of cyclic strain, not only do cells reorient away from the cyclic loading direction (Fig. 1a), but the distributions of shapes they assume also become more peaked (18,19). Associated with the reorientation of cells is also a reorientation of the SF arrangements whose angular distributions are typically quantified via circular histograms (Fig. 1a). The vast majority of models developed to understand cyclic strain avoidance include only the SF reorganization and ignore the observation that cell morphology and SF distributions are closely linked. Nevertheless, these models [e.g. Deshpande et al. (20), Vernerey and Farsad (21)], are successful in predicting cyclic strain avoidance by SFs for cells on 2D substrates: this avoidance stems from the sensitivity of SF stresses to strain-rate. However, these models cannot capture the alignment of the SFs with the imposed strain in a 3D setting. A modified model by Obbink-Huizer et al. (22) is able to account for the cyclic response of cells in both the 2D and 3D settings by including a strain dependence in the SF kinetics. However, the strain dependence of SF kinetics is hard to justify given the extensive remodeling that occurs on timescales of interest. To alleviate these issues, Vigliotti et al. (23) proposed a thermodynamically motivated model with the key feature that the cell strain-rate leads to the SF remodeling by concurrently adapting the SF angular distribution and the density of functional units in SFs. While this framework successfully predicts a range of observations, it again makes no reference to cell morphology and thus fails to include the coupled dynamics of SFs and cell morphology, which is essential to capture the cellular strain avoidance.

The coarse-grained model of Safran and colleagues (24,25) attempted to rationalize the reorientation of cells under applied strain. Specifically, they treated the cells as needles with the SFs within the needles acting as force dipoles. They hypothesized that this force dipole orients away from the imposed external stress to reduce the free energy of the system. However, since this coarse-grained approach only makes tenuous connections of the force dipoles with the intracellular structures, it is unable to predict the frequency-dependent SF organizations. Nevertheless, to the best of the authors’ knowledge, this model is the only approach in the literature that recognizes that SF orientations are intimately connected to cell orientation.

The coupled dynamics of the cell morphology and associated SF organization as a function of extra-cellular environment is a complex problem that received little attention. Recently, Shishvan et al. (26) proposed a statistical framework, called the homeostatic ensemble, that captures the interplay between the cytoskeletal structure and cell morphology. The approach has been shown to accurately predict the distribution of observed shapes of cells, in absence of cyclic loads, in numerous environments (6,8,27). Here, we extend this framework to cyclic loading conditions. We show, in quantitative agreement with observations, that SF distributions within a cell are not strongly affected by cyclic strain but rather cells preferentially reorient to avoid the cyclic strain direction. This alignment occurs by cell rotation rather than by stretching of the cell. These predictions are a consequence of the changes in the free-energy landscape for cells on 2D substrates subjected to cyclic strain: SFs subjected to contractile strain-rates exert lower stresses resulting in an increase in the free energy, which, in turn, results in cell reorienting to avoid contractile strain-rates.

## Dynamic equilibrium under cyclic strain

We consider a system comprising a cell adhered to an elastic substrate immersed in a nutrient bath at constant pressure and temperature and the substrate is subjected to cyclic strain (Fig. 1b). Typically, in such experiments (14), a dynamic steady state or equilibrium is attained after cycling for ∼12 hours, and the statistics of key observables such as cell area, shape, and orientations as well as SF arrangements within the cell reach steady state. We first develop a model to estimate the statistics of these observables after steady-state conditions under cyclic strain are achieved.

Cells respond to extracellular cues, such as cyclic strain, through cytoskeletal reorganization. The response of this complex system is recorded through a range of observables, all of which exhibit large variations (18,19). However, clear trends emerge when the statistics of these observables are analyzed. The homeostatic ensemble (26) has been shown to successfully predict these statistics for cells in a range of environments when no external loads are imposed (6,8,27). This motivates us to extend the framework to predict the response of cells on substrates subjected to cyclic loading.

### Cyclic homeostatic ensemble

Here, we briefly describe the cyclic homeostatic ensemble, with full details and mathematical derivations given in the Supplementary Material Section S1. The homeostatic ensemble recognizes that the cell is an open system that exchanges nutrients, such as glucose, Na^+^ ions, and oxygen with the surrounding nutrient bath (Fig. 1b). These high-energy nutrient exchanges cause large fluctuations (much larger than thermal fluctuations) in the cell response resulting from the various intracellular biochemical processes that are fueled by these nutrients. The fluctuations alter the cell morphology and the homeostatic ensemble predicts the distributions of states the system (the system is defined as the cell and substrate but the nutrient bath is excluded) assumes; see Supplementary Material Section S1. Specifically, the homeostatic ensemble defines a morphological microstate of the system recognizing that biochemical processes such as actin polymerization and treadmilling provide the mechanisms for the cell to explore morphological microstates. Then employing *ansatz* that the these processes result in the system maximizing the morphological entropy, the homeostatic ensemble provides the distribution of states the system (and the cells) attain in a given environment. The key constraint while maximizing the entropy is that the cells attain a homeostatic state, viz. the average number of all species within the cell is fixed independent of the environment. This constraint in fact is equivalent to the statement that cells explore a range of morphological states with a fixed energy “budget.”

In broad terms, a morphological microstate specifies the shape of a cell. More specifically, a morphological microstate is defined by the mapping (connection) of material points on the cell membrane to the material points on the substrate (Fig. 1b). An important assumption in developing the cyclic homeostasis framework is a separation of time scales. There are three relevant timescales in the problem.

1. *Time for the intracellular processes*, $T_{\textrm {intra}}$. This is driven by a range of biochemical processes, including cytoskeletal processes such as actin polymerization, myosin power strokes driving SF contraction, and diffusion of species such as unbound cytoskeletal and signaling proteins within the cell. These processes are relatively fast and are typically limited by diffusion rates (chemical reactions and mechanical processes, such as wave propagation are typically much faster and thus not the rate-limiting processes) (28). Therefore, intracellular remodeling occurs on the order of a few seconds, i.e. $T_{\textrm {intra}} = \mathcal {O}(1~\textrm {s})$.
2. *Time for the evolution of morphological microstates*, *T*_cell_. Evolution of a morphological microstate or cell morphology requires co-operative cytoskeletal processes within the cell, such as cytoskeletal reorganization orchestrated by coordinated actin polymerization, treadmilling, and dendritic nucleation (29–31). These cytoskeletal processes are much slower and thus cell morphology evolves slowly and on the timescale of minutes, i.e. $T_{\textrm {cell}}= \mathcal {O}(1~\textrm {min})$. This, in turn, implies $T_{\textrm {cell}} \gg T_{\textrm {intra}}$.
3. *Time period T*_p_ *of cyclic straining*. We will focus on the cyclic strain with a time period $T_{\textrm {p}} \ll T_{\textrm {cell}}$. Given that cyclic strain conditions of primary interest are typically around the physiologically relevant frequency of 1 Hz, this covers most realistic cyclic straining conditions.

Thus, the three timescales are related by

(1) $$
\begin{equation*}
T_{\textrm {intra}} \sim T_{\textrm {p}}\ll T_{\textrm {cell}}.
\end{equation*}
$$

Similar separation of timescales is also used in refs. (24,32) and reported in experiments (33, 34), where the SFs reorient appreciably faster compared to the cell (while the case of $T_{\textrm {p}} \ll T_{\textrm {intra}}$ is beyond the scope of the model, we anticipate that when $T_{\textrm {p}} \sim T_{\textrm {cell}}$, alignment under cyclic loading is expected to be lost as the imposed frequencies are less than 0.01 Hz.). The separation of timescales allows us to assume that for a given cell morphology (or morphological microstate), the intracellular structure is well approximated by its steady-state configuration. In addition, during a time period of cyclic straining, the morphological microstate undergoes a negligible change. Exploiting these assumptions, we show (Supplementary Material Section S1.4) that the equilibrium probability of a morphological microstate (*c*) under cyclic loading conditions is given by

(2) $$
\begin{equation*}
P^{(c)}_{\textrm {eq}}= \frac{\textrm {exp}(-\beta (H^{(c)}-\phi ^{(c)}))}{\sum _c \textrm {exp}(-\beta (H^{(c)}-\phi ^{(c)}))},
\end{equation*}
$$

where *H*^(*c*)^ is the time-averaged Helmholtz free energy of the system over period *T*_p_ and Φ^(*c*)^ is a term associated with the elastic deformation of the substrate due to the tractions exerted on the substrate by the adherent cell. The distribution parameter β is set by the cellular homeostatic constraint, viz. over all the fluctuations of the cell, the numbers of each species (Na^+^, Glucose) within the cell remains at fixed values, independent of the extracellular environment/loading. This constraint reduces to (Supplementary Material Section S1.4)

(3) $$
\begin{equation*}
\sum _{c} P^{(c)}_{\textrm {eq}}(H^{(c)}-\phi ^{(c)}) = H_\textrm {s},
\end{equation*}
$$

where *H*_s_ is the Helmholtz free energy of the cell in suspension (i.e. the unadhered cell). The distribution $P^{(c)}_{\textrm {eq}}$ is dependent on the imposed cyclic strain profile and provides the statistics of all the observables for the steady or equilibrium state of the cell under cyclic loading conditions.

While the elastic properties of cyclically strained substrates are known to influence the organization of cells (35–37), in many reported experiments (14,35,38), substrates are “stiff” (e.g. silicone) compared to cells such that the tractions exerted by the cell on the substrate result in negligible substrate deformation. The advantage of such substrates is that over the period *T*_P_, the imposed cyclic strains (and strain-rates) are directly transmitted to the cell membrane adhered to the substrate and this simplifies the interpretation of the measurements. We restrict our analysis to this “stiff” limit and will subsequently show that it suffices to accurately predict numerous reported observations. In the stiff limit, we can neglect the substrate strain due to cell tractions and focus on the cyclic response of the cells. Using this assumption, we show in Supplementary Material Section S1.5 that Φ^(*c*)^ is the average substrate elastic energy that is independent of the morphological microstate (*c*) and depends only on the imposed strain profile. It then straightforwardly follows that $H^{(c)}-\phi ^{(c)}=H^{(c)}_{\textrm {cell}}$, where $H^{(c)}_{\textrm {cell}}$ is the time-averaged Helmholtz free energy of the cell over the period *T*_p_. Consequently, Eqs. 2 and 3 reduce to

(4) $$
\begin{equation*}
P^{(c)}_{\textrm {eq}}= \frac{\textrm {exp}\big(-\beta H_{\textrm {cell}}^{(c)}\big)}{\sum _c \textrm {exp}\big(-\beta H_{\textrm {cell}}^{(c)}\big)},
\end{equation*}
$$

with

(5) $$
\begin{equation*}
\sum _{c} P^{(c)}_{\textrm {eq}}H^{(c)}_{\textrm {cell}} = H_{\textrm {s}}.
\end{equation*}
$$

Thus, the morphological microstates the cell assumes are independent of the elastic properties of the substrate.

### Morphological microstate and the free-energy $\mathbf{H}_{\mathbf{cell}}^{(\mathbf{c})}$

A morphological microstate is defined by the connection of material points on cell membrane to material points on the substrate. Cells take a large number of complex 3D shapes, but to reduce the computational cost, Deshpande and co-workers (6,8, 26) have shown that in a number of situations it is sufficient to approximate the cells as 2D bodies as shown in Fig. 1c. In these works, nonuniform rational B-splines are used to describe complex 2D cells and morphological metrics such as cell area, aspect ratio are extracted in a manner similar to that reported in the majority of experimental studies. In extracting such metrics, cells are often approximated as ellipses and thus here, we simplify the computational approach further by approximating cells as spatially uniform ellipses in the plane of substrate. We expect that while this approximation will miss some features, it will suffice to capture key morphological observables (area, aspect ratio, and cell orientation) of interest.

Consider a spatially uniform ellipse on a flat substrate in the ${\mathbf{ x}}_1\hbox {--}{\mathbf{ x}}_2$ (Fig. 1d). The morphological microstate is then naturally characterized by the area *A* = π*a*_1_*a*_2_, aspect ratio *A*_*s*_ = *a*_1_/*a*_2_ ≥ 1, and the orientation θ of the major axis of the ellipse with respect to the ${\mathbf{ x}}_1$-direction, with *a*_1_ and *a*_2_ the lengths of the semi-major and semi-minor axes of the ellipse, respectively. In our statistical mechanics framework, the cell samples a phase space comprising variables that describe the morphological microstate. It is thus preferable to use microstate variables that span similar extents and we thus use the analytic geometry definition of the ellipse to define a morphological microstate. Further, we restrict ourselves to the case of homogeneous substrate where the energy of the system is independent of the cell location on the substrate and therefore it suffices to describe the cell morphology by an ellipse with its centroid fixed. Then, the points on the periphery of the ellipse with centroid located at (*x*_1_, *x*_2_) = (0, 0) satisfy the implicit equation 

(6) $$
\begin{equation*}
h\hat{x}_1^2+k\hat{x}_1\hat{x}_2+l\hat{x}_2^2=1,
\end{equation*}
$$

where $\hat{x}_1=x_1/R_0$ and $\hat{x}_2=x_2/R_0$ with *R*_0_ an arbitrary length scale that subsequently we will associate with the size of the cell in a reference state, while (*h*, *k*, and *l*) are nondimensional coefficients that describe the ellipse. These coefficients are related to (*a*_1_, *a*_2_, and θ) by

(7) $$
\begin{equation*}
h = \frac{\textrm {cos}^2(\theta )}{\hat{a}_1^2}+\frac{\textrm {sin}^2(\theta )}{\hat{a}_2^2},\quad k=\Bigg (\frac{1}{\hat{a}^2_1}-\frac{1}{\hat{a}^2_2}\Bigg )\textrm {sin}(2\theta ),~\textrm {and}\nonumber\\
l = \frac{\textrm {cos}^2(\theta )}{\hat{a}_2^2}+\frac{\textrm {sin}^2(\theta )}{\hat{a}_1^2},
\end{equation*}
$$

where $\hat{a}_1=a_1/R_0$ and $\hat{a}_2=a_2/R_0$.

The free-energy *H*^(*c*)^ is dependent on the steady-state intracellular structure for the given cell morphology (*c*) and the imposed cyclic loading. Modeling all the intracellular elements is unrealistic and might not provide appropriate physical insight. Moreover, it is well-known that the acto-myosin SFs remodel to dictate the cellular response under cyclic straining. Thus, we implement a relatively simple model to capture the active mechano-bio-chemistry of the SFs (23). The model is described in detail in Supplementary Material Section S2 and comprises contributions from the passive elasticity of the cytoplasm and nucleus as well as the active response of the acto-myosin SFs.

The cell in its undeformed state (also known as the elastic resting state since the elastic strain energy is zero in this state) is a circle of radius *R*_0_ and includes a circular nucleus of radius *R*_*N*_ whose centroid coincides with that of the cell. The cytoplasm is modeled as comprising of an active SF cytoskeleton wherein the actin and myosin proteins exist either in unbound or in polymerized states (Fig. 1c). Recall that the morphological microstate of the cell is described by (*h*, *k*, and *l*) such that the cell deforms to form an ellipse with a spatially uniform strain distribution. Thus, while the temporal mean strain of the cell with morphology (*c*) is specified by (*h*, *k*, and *l*), the strain-rate equals the substrate strain-rate since the cell remains adhered to the substrate within the period *T*_p_. The polymerized SF cytoskeleton is modeled as a distribution of active contractile SFs such that $\hat{\eta }(\phi )$ parameterizes the angular concentration of the SFs at angle ϕ, measured relative to the major axis of the ellipse, with $\hat{n}(\phi )$ denoting the number of functional units within each SF. Then, the total concentration $\hat{N}_b$ of bound SF proteins is obtained by integrating $\hat{\eta }\hat{n}$ over all orientations ϕ and the remaining proteins with a concentration $\hat{N}_u=1-\hat{N}_b$ remaining in the unbounded state. The angular distribution and chemical potentials of the bound proteins along with the concentration of the unbound proteins together provide the cytoskeletal free-energy *H*_cyto_(*t*) at time *t* within the period *T*_p_. Denoting the contribution from the lumped elasticity of the passive elements within the cytoplasm and nucleus by *H*_passive_(*t*), the Helmholtz free energy at time *t* for the cellular morphology (*h*, *k*, and *l*) is

(8) $$
\begin{equation*}
H_{\textrm {cell}}(t)=H_{\textrm {cyto}}(t)+H_{\textrm {passive}}(t).
\end{equation*}
$$

The time average over the period *T*_p_ then defines $H^{(c)}_{\textrm {cell}}$ in Eq. 4 for the morphological microstate (*c*), i.e.

(9) $$
\begin{equation*}
H^{(c)}_{\textrm {cell}}=\frac{1}{T_{\textrm {p}}}\int _{t_I}^{t_I+T_{\textrm {p}}}H_{\textrm {cell}}(t) dt,
\end{equation*}
$$

where *t*_*I*_ is an arbitrary time that defines the initial condition of the period over which the averaging is performed. The numerical procedure to calculate the cyclic energy is described in Supplementary Material Section S4.1.

While details for the free-energy model are provided in Supplementary Material Section S2.1, it is worth summarizing two key features of the model that determine cell behavior under cyclic straining:

1. Polymerization of SF proteins associated with cell spreading and elongation reduces *H*_cyto_. Thus, elongated and spread cell shape are preferred until it becomes energetically unfavorable due to the higher elastic strain energy *H*_passive_ associated with these shapes.
2. The stress in an SF is given by a Hill-type constitutive relation (Supplementary Fig. S1). Since the tensile SF stress decreases with increasing contraction rate, the SF free-energy *H*_cyto_ increases with increasing contraction rate.

These two features imply that while cells prefer to assume elongated shapes, cells elongated along the cyclic straining direction have a higher free energy compared to the same cell shape aligned along a direction where the SFs are subjected to lower strain-rates, as will be shown in the next section. In fact, we shall subsequently show that the cell free-energy $H^{(c)}_{\textrm {cell}}$ continuously decreases as an elongated cell of fixed shape rotates away from the cyclic strain direction.

## Predictions of the cyclic steady state

We present predictions of the steady state that cells assume when subjected to cyclic straining on a stiff substrate. This steady state is given by probability distribution Eq. 4 and typically observed after the cells have been subjected to cyclic loading for  12 hours or more. In line with most experiments, we consider cyclic straining of the substrate such that the principal strains ε_1_(*t*) and ε_2_(*t*) are given by

(10) $$
\begin{equation*}
\varepsilon _1(t)=\varepsilon _\textrm {mean}+\frac{\varepsilon _{\textrm {amp}}}{2} \textrm {sin}(2\pi f t),
\end{equation*}
$$

and ε_2_ = −*r*ε_1_, where *r* is a measure of the biaxiality of the imposed cyclic strain. Then the substrate principal stretches are λ_1_(*t*) = 1 + ε_1_(*t*) and λ_2_(*t*) = 1 + ε_2_(*t*), while ε_mean_ is the mean strain with ε_amp_ the peak-to-peak amplitude of the imposed cyclic strain at frequency *f*. Unless otherwise specified, we restrict the results to the case of uniaxial straining with *r* = 0 and only show a few predictions for the range 0 < *r* ≤ 1 to demonstrate the generality of the model. For stiff substrates, the morphological microstate (*c*) is independent of the mean substrate strain ε_mean_ and hence the cyclic results are independent of ε_mean_ (and thus not specified here). The numerical procedure to compute the distribution of observables at the cyclic steady state is summarized in Supplementary Material Section S4.2. The majority of the cyclic results presented here are for the physiologically relevant parameters that are used extensively in experiments reported in the literature, viz. ε_amp_ = 0.1 and *f* = 1 Hz (14, 18), with material parameters to determine active and passive energies provided in Supplementary Tables S1 and S2. A more extensive parametric study is reported in Supplementary Material Section S5.

### Effects of cyclic strain on the dynamic equilibrium of cells

Typically in experiments, the effect of cyclic strain on the SF angular distribution is of primary interest. To characterize the SF distributions in our results, we define the metric

(11) $$
\begin{equation*}
\xi (\delta )=\sum _{c}P^{(c)}_{\textrm {eq}}\hat{\eta }(\phi )\hat{n}(\phi ).
\end{equation*}
$$

**Fig. 2.**
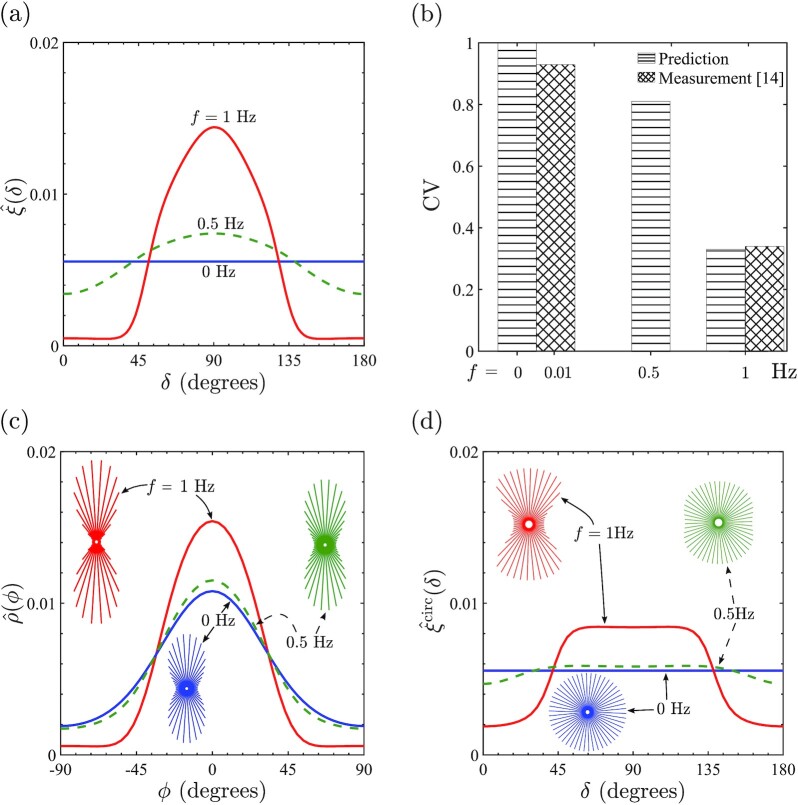
(a) Predictions of the angular distributions of SF concentrations as parameterized by $\hat{\xi }(\delta )$, where δ is the orientation of the SFs with respect to ${\mathbf{ x}}_1$-direction of cyclic stretching. Results are shown for cyclic loading (*r* = 0) with ε_amp_ = 0.1 and *f* = 0.5, 1 Hz, together with the reference case of no imposed cyclic loading (i.e. *f* = 0). (b) Comparison of the predicted and measured (14) CVs, defined by (12), for selected frequencies *f* and ε_amp_ = 0.1. (c) The angular distributions of the SF concentrations as parameterized by $\hat{\rho }(\phi )$ within cell with *ϕ* denoting the orientation of the SFs with respect to the major axis of the ellipse. Predictions are shown for the three straining cases in (a) with the corresponding circular histograms shown as insets. (d) The angular distributions and circular histograms for SFs parameterized by $\hat{\xi }^{\textrm {circ}}(\delta )$ for a circular cell of radius *R*_0_ and subjected to the three straining cases in (a).

Here, the product $\hat{\eta }(\phi )\hat{n}(\phi )$ provides a measure of actin concentration (see Supplementary Material Section S2.1) in morphological microstate (*c*) at an orientation ϕ = δ − θ with respect to the major axis of the ellipse with δ the angle of the SF with respect to $\mathbf {x}_1$-direction (Fig. 1e). Thus, ξ provides the ensemble average of the SF concentration at an orientation δ over all morphological microstates in the cyclic homeostatic ensemble. Predictions of the $\hat{\xi }(\delta )=\xi (\delta )/\int _{-\pi /2}^{\pi /2}\xi (\delta ) d\delta$ are shown in Fig. 2a for a cyclic strain (*r* = 0) with ε_amp_ = 0.1 and *f* = 0.5, 1 Hz along with the reference case of no imposed cyclic strain (i.e. *f* = 0 Hz). While $\hat{\xi }$ is isotropic for *f* = 0 Hz, there is a strong tendency for SFs, as parameterized by $\hat{\xi }$, to be preferentially orientated at δ = 90^○^ with respect to the cyclic strain direction. Consistent with observations (14), the tendency of the SFs to “avoid” the cyclic strain direction increases with increasing frequency of the cyclic strain; see Supplementary Fig. S2 in Supplementary Material Section S5 for a more detailed parametric study, including dependence on strain amplitude. To make a direct comparison with metrics reported in measurements (14), we compute the circular variance (CV) of $\hat{\xi }$ defined as

(12) $$
\begin{equation*}
\textrm {CV}=1-\sqrt{\left[\int _0^\pi \hat{\xi }(\delta )\cos (2\delta ) d\delta \right]^2+\left[\int _0^\pi \hat{\xi }(\delta )\sin (2\delta ) d\delta \right]^2}.
\end{equation*}
$$

Comparisons between measurements (14) and predictions shown in Fig. 2(b) indicate remarkable agreement with measurements (14) for *f* = 1 Hz. Of course, consistent with the distributions of $\hat{\xi }$ in Fig. 2(a), CV increases with decreasing frequency and attains the isotropic value of CV = 1.0 at *f* = 0 Hz.

While Fig. 2(a) and (b) clearly shows that with no cyclic strain, there is no orientational bias of the SFs with respect to the ${\mathbf{ x}}_1$-direction, it is well established that in the absence of cyclic strain cells seeded on stiff substrates assume elongated shapes with aligned SFs (3,14). To investigate the alignment of SFs within cells, we define a parameter analogous to $\hat{\xi }$, viz. $\rho (\phi )=\sum _{c}P^{(c)}_{\textrm {eq}}\hat{\eta }(\phi )\hat{n}(\phi )$, with $\hat{\rho }(\phi )=\rho /\int _{-\pi /2}^{\pi /2}\rho d\phi$. Predictions of $\hat{\rho }$ in the form of an x–y plot and circular histograms are shown in Fig. 2(c) for cyclic strain with ε_amp_ = 0.1 and *f* = 0.5, 1 Hz along with the no cyclic strain case of *f* = 0 Hz. The differences between the three cases now are much less pronounced with strong alignment of the SFs along the major axis of the ellipse (*ϕ* = 0) seen in all cases, although the level of alignment does marginally increase with increasing *f*. The circular variance of $\hat{\rho }$ can be defined analogously to Eq. 12 and are 0.61, 0.57, and 0.33, respectively, for *f* = 0, 0.5, and 1 Hz.

**Fig. 3.**
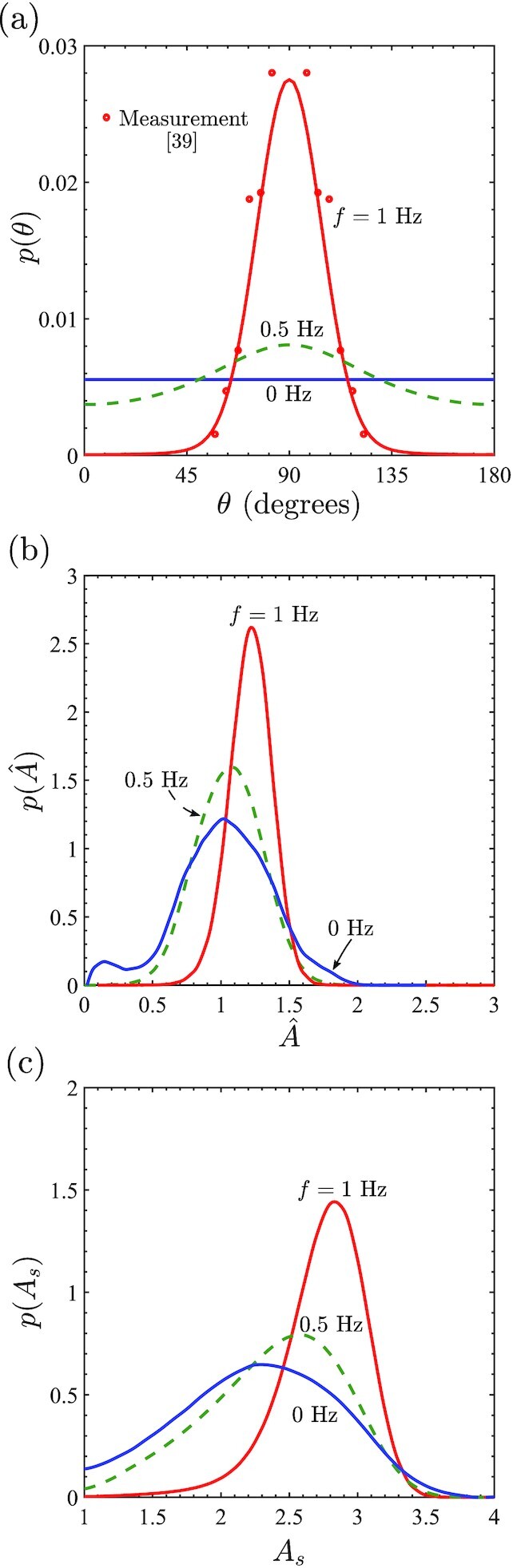
Probability density functions of the three key morphological observables for loading (*r* = 0) with a cyclic strain ε_amp_ = 0.1 and *f* = 0.5, 1 Hz along with the case of no cyclic strain (*f* = 0 Hz). (a) Predictions of the cell orientation *p*(θ) along with comparisons with measurements of Wang et al. (39) for endothelial cells subjected to uniaxial cyclic strain with *f* = 1 Hz. Corresponding predictions of the (b) normalized cell area $p(\hat{A})$ and (c) cell aspect ratio *p*(*A*_*s*_).

Cyclic strain has a small effect on SF alignment with respect to the cell morphology but a strong influence on the angular distribution of SFs with respect to the global ${\mathbf{ x}}_1$-direction. This suggests that cyclic strain strongly influences the orientation θ of the cells. Predictions of the probability density of θ for ε_amp_ = 0.1 and *f* = 0.5, 1 Hz with *r* = 0 along with the no cyclic strain case of *f* = 0 Hz are included in Fig. 3(a). While consistent with experimental observations (18,19), there is no preferential orientation of the cells for *f* = 0 Hz, cells align perpendicular to the cyclic strain direction ${\mathbf{ x}}_1$ with the degree of alignment increasing with increasing *f*. We also include in Fig. 3(a) the quantitative comparisons between predictions and measurements (39) [which we have symmetrized to extend in range to (0 to 180^○^)] for the *f* = 1 Hz case. The excellent agreement with measurements demonstrates the fidelity of the predictions. The SF alignments within cells (Fig. 2c) and cell alignments (Fig. 3a) together clearly show that SF alignment under cyclic strain away from the cyclic strain direction (Fig. 2a and b) is primarily a consequence of cells being preferentially oriented perpendicular to the cyclic strain direction rather than a significant change to the SF arrangements within cells.

Cyclic strain is also known to alter cell morphology with observations showing that cell aspect ratio increases with the cyclic strain frequency (18,19, 40). Predictions of the probability density distributions of normalized cell area $\hat{A}=A/(\pi R_0^2)$ and aspect ratio *A*_*s*_ are shown in Fig. 3(b) and (c), respectively, for cyclic strain with ε_amp_ = 0.1 and *f* = 0.5, 1 Hz along with the reference case of *f* = 0 Hz. Our model not only predicts an increase in the mean area and aspect ratio of the cells under cyclic strain, but more importantly it predicts that narrower probability distributions (i.e. less variability in cell morphology) under cyclic strain, similar to observations of Greiner et al. (18).

Our predictions indicate that cyclic strain mainly influences cell orientation (and to a lesser extent cell morphology) rather than SF alignments within cells. While these predictions are consistent with experimental observations, they contrast with all existing models (20,21,23) for the effect of cyclic strain on cells. These models all attribute the main effect of cyclic strain to be on SF alignment within cells rather than on cell orientation. The reason for this is that the existing models are restricted to modeling the SF arrangements within cells with no consideration of cell morphology. In effect, such previous modeling approaches implicitly assume a circular cell and investigate the influence of cyclic strain on SF arrangements for this fixed circular morphology where cell orientation is not a relevant parameter. Such models only require a framework for SF remodeling with no connection to simultaneously estimate cell morphology. In contrast, our cyclic homeostasis framework predicts the influence of cyclic strain on cell morphology by connecting cell morphology to the SF arrangements under cyclic straining. In the cyclic homeostatic framework, it is possible to decouple cell morphology and SF remodeling to investigate the influence of cell morphology on SF arrangements. We thus restrict the cell to circular with radius *R*_0_ and just employ the SF model (see the “Morphological microstate and the free-energy $H^{(c)}_{\textrm {cell}}$” section  and Supplementary Material Section S2) to investigate the influence of cyclic straining on SF arrangements. This gives predictions that are directly comparable to existing models (20,21,23). With cell morphology fixed to be circular, $\hat{\xi }$ and $\hat{\rho }$ are identical parameters and both reduce to $\hat{\xi }^{\textrm {circ}}(\delta )=\hat{n}(\delta )\hat{\eta }(\delta )/\int _{-\pi /2}^{\pi /2}\hat{n}(\delta )\hat{\eta }(\delta ) d\delta$, where δ = ϕ. Circular histograms and XY plots and of $\hat{\xi }^{\textrm {circ}}(\delta )$ are included in Fig. 2(d) for ε_amp_ = 0.1 and *f* = 0.5, 1 Hz along with the case of *f* = 0 Hz. This simple circular cell model, which only accounts for changes in SF arrangements due to cyclic straining, predicts circular histograms that are qualitatively consistent with the predictions of the cyclic homeostasis in Fig. 2(b), but quantitatively they are quite different. To clarify this, we define CV^circ^ analogously to Eq. 12 with $\hat{\xi }$ replaced by $\hat{\xi }^{\textrm {circ}}$. For *f* = 1 Hz, CV^circ^ = 0.90, which is significantly higher than the measured value of 0.34, i.e. restricting the cell to be circular predicts a reduced level of SF alignment. Thus, the circular cell model is inconsistent with experimental observations in two important aspects: it predicts (i) that the SF distributions within a cell on a substrate not subjected to cyclic strain is isotropic and (ii) the key effect of cyclic strain is on SF alignment within cells rather than on the orientation of cells. A corollary consequence of (ii) is that it predicts a significantly lower level of SF alignment under cyclic straining in comparison to experimental measurements.

### Changes in the free-energy landscape drive the changes in cell morphology and orientation

**Fig. 4.**
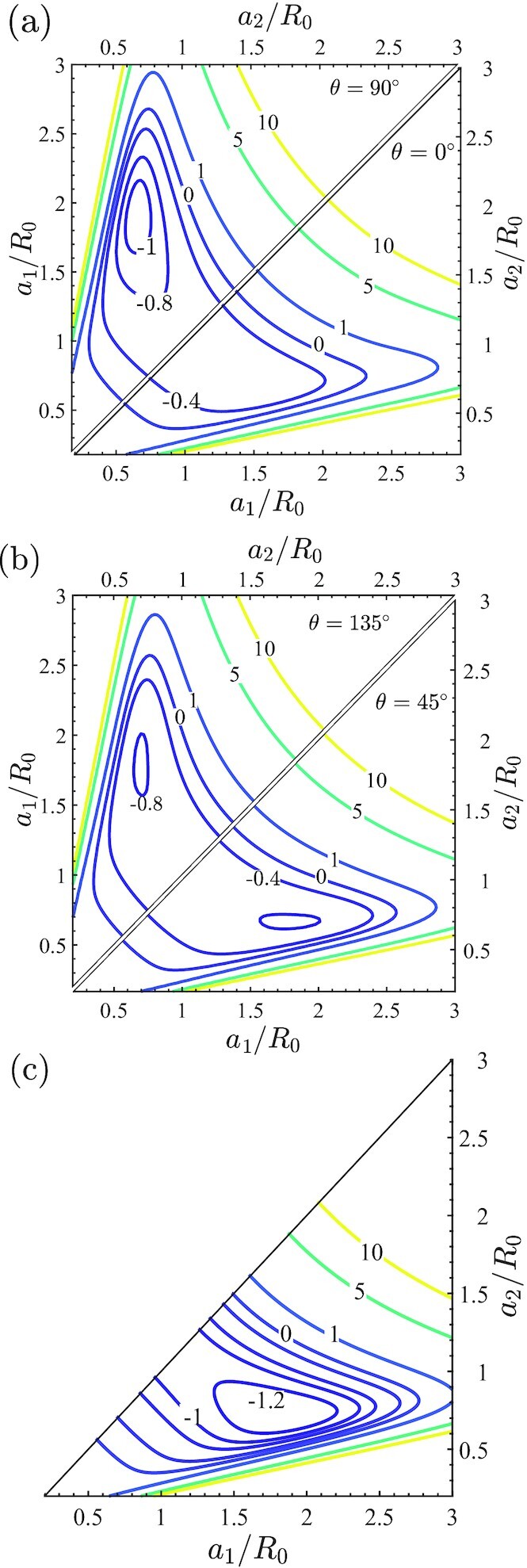
Predictions of the normalized free-energy $\hat{H}=H^{(c)}_{\textrm {cell}}/|H_{\textrm {s}}|$ landscapes using axes of the normalized semi-major and semi-minor axes *a*_1_/*R*_0_ and *a*_2_/*R*_0_, respectively, of the ellipse. These landscapes are shown for cyclic strain (*r* = 0) with ε_amp_ = 0.1 and *f* = 1 Hz for cells oriented at (a) θ = 0^○^ and 90^○^, (b) θ = 45^○^ and 135^○^, and (c) the reference case of no imposed cyclic strain (i.e., *f* = 0 Hz). In (c), we only show the landscape for *a*_1_ ≥ *a*_2_, where *a*_1_ is the semi-major axis and there is no θ dependence of the free-energy landscape.

The cyclic homeostatic framework makes predictions consistent with a range of experimental observations. Recalling that $P_{\textrm {eq}}^{(c)}$ is set by $H_{\textrm {cell}}^{(c)}$ in Eq. 4, the landscapes of $H_{\textrm {cell}}^{(c)}$ in the morphological phase space provide insights into the predictions reported above. Using axes of *a*_1_/*R*_0_ and *a*_2_/*R*_0_, we show the landscapes of the normalized Helmholtz free-energy $\hat{H}=H_{\textrm {cell}}^{(c)}/|H_{\textrm {s}}|$ for cell orientations θ = 0^○^, 90^○^, and 45^○^, 135^○^ in Fig. 4(a) and (b), respectively, for cyclic straining with ε_amp_ = 0.1 and *f* = 1 Hz. [Note that while $H_{\textrm {cell}}^{(c)}$ is presented here as a function of the physically intuitive geometrical parameters *a*_1_/*R*_0_, *a*_2_/*R*_0_, and θ, $P_{\textrm {eq}}^{(c)}$ is estimated by sampling the morphological phase space in terms of (*h*, *k*, and *l*).] These landscapes will help interpret three key predictions, viz. (i) cells reorient away from the cyclic strain direction; (ii) the shapes they assume, and (iii) the narrowing of the probability distributions of the cell morphologies under cyclic strain.

First, compare the θ = 0^○^, 90^○^ landscapes in Fig. 4(a). Clearly, overall $\hat{H}$ values are lower for θ = 90^○^ compared to θ = 0^○^ and a local minimum for $\hat{H}$ is found for the θ = 90^○^ case at (*a*_1_/*R*_0_, *a*_2_/*R*_0_) ≈ (1.9, 0.68). The lower values of $\hat{H}$ for nearly all cell morphologies with θ = 90^○^ compared to θ = 0^○^ implies that at dynamic equilibrium, θ = 90^○^ morphologies have a higher probability to be observed as seen in the predictions on Fig. 3(a). The θ = 45^○^ and 135^○^ landscapes in Fig. 4(b) show that at these orientations, $\hat{H}$ values are intermediate to the θ = 0^○^, 90^○^ cases resulting in intermediate values of the probability at those orientations. To understand the differences in the free-energy landscapes at different cell orientations, recall that SF polymerization as characterized by $\hat{\eta }\hat{n}$ is maximum in the direction of maximum cell strain. For the θ = 90^○^, this direction is perpendicular to the cyclic strain with these fibers close to isometric conditions and hence under high tensile stress. This leads to these fibers having a low enthalpy that translates to a low $H_{\textrm {cell}}^{(c)}$ (see Supplementary Material Section S2.1). On the other hand, for the same elliptical shape but with θ = 0^○^, the fibers along the direction of maximum polymerization are subject to a high contractile strain-rate during the contractile phase of cyclic straining. These contractile strain-rates reduce the fiber stress via a Hill-type relation and increase their enthalpy and thereby increase $H_{\textrm {cell}}^{(c)}$.

The morphologies cells adopt and the effect of cyclic strain on cell morphologies are best considered together. The free-energy landscape $\hat{H}$ in the absence of cyclic strain is shown in Fig. 4(c). Of course, cell orientation no longer plays a role in this case with the landscape independent of θ. The minimum in $\hat{H}$ at (*a*_1_/*R*_0_, *a*_2_/*R*_0_) ≈ (1.85, 0.72) sets the mode of the area and aspect ratio probability distributions (in the absence of cyclic strain) plotted in Fig. 3(b) and (c), respectively. To understand this minimum, recall that $H_{\textrm {cyto}}^{(c)}$ decreases with increasing cell spreading due to higher levels of polymerization. However, cell spreading also increases the elastic energy of the cell and the two together compete to give the minima [see Fig. 7 of (8)]. The spreading is not isotropic but results in elongated cell morphologies as the shear modulus of the cell is lower than its bulk modulus and thus from an elastic standpoint, it is energetically favorable to assume elongated spread shapes. These basic phenomena are also at play under cyclic strain (see replot of the free-energy landscapes in Supplementary Fig. S3 with contours of cell area and aspect ratio included) and hence the free-energy landscape under cyclic strain with θ = 90^○^ (Fig. 4a) and in the absence of cyclic strain (Fig. 4c) are qualitatively similar. So why do cell morphologies become more deterministic under cyclic strain? Notice that the free-energy landscapes for the cyclic θ = 90^○^ case has a more localized region of low $\hat{H}$, i.e. the free-energy well is confined over a smaller region of the morphological phase space compared to in the absence of cyclic strain. Therefore, to satisfy the homeostatic constraint under cyclic strain, the probabilities of these low free-energy states need to be higher relative to the higher free-energy states with the consequence that the cells adopt a smaller variation of morphologies under cyclic strain. As a corollary, the system also acquires larger value of the distribution parameter β under cyclic strain (β|*H*_s_| = 18.30 for cyclic straining with ε_amp_ = 0.1 and *f* = 1 Hz, while β|*H*_s_| = 5.80 in the absence of cyclic straining). Analogous to the usual canonical ensemble, the distribution parameter β can be viewed as the inverse of the “homeostatic temperature” and thus cyclic straining reduces the homeostatic temperature and therefore makes the cell more deterministic.

### Cells align along the direction of vanishing cyclic strain-rate

The above uniaxial cyclic straining (*r* = 0) results suggest that cells primarily align along the direction of zero strain-rate. In this orientation, the majority of SFs are under isometric conditions and this reduces the overall free energy of the cell. To check the generality of this prediction, we now consider biaxial cyclic loading of the substrate with 0 ≤ *r* ≤ 1. Keeping in mind that our aim is to predict the orientation of the cells, we now employ a 1D (or rod-like model) for the cell rather than modeling cells as ellipses. Spatially uniform rod-like cells implies that the microstate (*c*) of the cell is modeled by just two degrees of freedom, the cell stretch λ, and cell orientation θ; see Supplementary Material Section S3 for details. This approximation substantially reduces the numerical cost of the model and suffices to describe distribution of the cell orientations. When this rod-like model for cells is implemented within the statistical framework, it provides predictions for the probability distribution of cell orientations (Supplementary Fig. S4a for *f* = 1 Hz and ε_amp_ = 0.1) for each choice of the loading biaxiality *r*. For *r* > 0 two modes are observed in the probability distributions at orientations of vanishing strain-rate, viz. $\theta =\tan ^{-1}(1/\sqrt{r})$ and $\theta =\pi -\tan ^{-1}(1/\sqrt{r})$. The reasons are the same as discussed earlier in the context of elliptical cells: in the direction of vanishing strain-rate, the SFs are under isometric conditions and this minimizes the cell free energy for a given value of λ. Comparisons between predictions of the mode of *p*(θ), viz. $\theta =\tan ^{-1}(1/\sqrt{r})$ and measurements (32) are included in Supplementary Fig. S4b. Excellent agreement with observations confirms that cells indeed to orient in directions of vanishing substrate strain-rate.

## Evolution of cell morphology under cyclic strain

Under dynamic equilibrium conditions, cells on substrates subjected to cyclic straining orient themselves away from the strain direction with the consequence that the SFs too are primarily aligned away from the cyclic strain direction. However, the dynamic equilibrium analysis discussed earlier did not address the mechanism of this process; viz. did cells reorient themselves away from the cyclic strain direction by rotating while keeping cell morphology fixed or stretch themselves so as to contract in the cyclic strain direction and elongate in other directions to ultimately result in reorientation? To answer this question, a kinetic analysis of the temporal evolution of the cells under cyclic strain is required.

### A Langevin formulation for cyclic strain

Within the context of the cyclic homeostatic ensemble for cells on stiff substrates, the free-energy $H^{(c)}_{\textrm {cell}}$ of the cell fluctuates under dynamic equilibrium conditions but the corresponding cyclic homeostatic potential *M* = *H*_s_ − (1/β)*S*_T_, where *S*_T_ is the morphological entropy of the cell, is constant over these fluctuations (Supplementary Material Section S1.5). This establishes a direct analogy between the homeostatic ensemble and the well-established canonical ensemble, where the system energy fluctuates but the Helmholtz free energy remains constant. Since the temporal evolution of the microstates of an isothermal system whose equilibrium distribution is given by the canonical ensemble is often described by the Langevin dynamics, we present a similar equation to characterize the kinetics of cell evolution. The low “speeds” at which cell morphologies fluctuate implies that it suffices to consider overdamped Langevin dynamics and ignore inertia. Such an approach was pursued by Ippolito et al. (8) for cells on substrates in the absence of cyclic strain and here we extend it to when substrate on which these cells are seeded is subjected to cyclic straining. Specifically, the Langevin equation describes the evolution of the cell morphology parameterized by the nondimensional coefficients (*h*, *k*, and *l*). The overdamped Langevin equation for the evolution of the cell morphology is then written as

(13) $$
\begin{equation*}
\frac{\partial r_i}{\partial t} = -\frac{1}{\gamma } \frac{\partial H_{\textrm {cell}}^{(c)}}{\partial r_i} + \sqrt{\frac{2}{\beta \gamma }}W(t),
\end{equation*}
$$

where $\mathbf {r}=(h,k,l)$, and γ is a damping coefficient, sometimes referred to as the mobility, and *W*(*t*) a Wiener process. Recall that our assumption of the separation of timescales implies that the coefficients $\mathbf {r}$ evolve over timescales ≫ the cyclic straining period *T*_p_ and the timescales over which the intracellular structure adapts. Thus, in Eq. 13, we employ the cyclic free-energy $H_{\textrm {cell}}^{(c)}$ for describing the temporal evolution of $\mathbf {r}$.

A key justification of the validity of this approach is that it recovers the dynamic equilibrium distribution discussed in the section “Cyclic homeostatic ensemble.” To observe this note that the Fokker–Planck equation corresponding to Eq. 13 is given by

(14) $$
\begin{equation*}
\frac{\partial P(\mathbf {r},t)}{\partial t}= \frac{1}{\gamma }\sum _{i=1}^{3}\frac{\partial }{\partial r_{i}}\Big (P(\mathbf {r},t)\frac{\partial H_{cell}^{(c)}}{\partial r_i}\Big ) + \frac{\beta }{\gamma } \sum _{i=1}^{3}\frac{\partial ^2 P(\mathbf {r},t)}{\partial^2 r_i},
\end{equation*}
$$

where $P(\mathbf {r},t)$ is the probability of morphological microstate (*c*) parameterized by $\mathbf {r}$ at time *t*. The steady-state solution to Eq. 14 corresponding to $\partial P(\mathbf {r},t)/\partial t = 0$ is the equilibrium probability distribution and is then

(15) $$
\begin{equation*}
P_{\textrm {eq}}(\mathbf {r})=\frac{1}{Z}\textrm {exp}\big(-\beta H_{\mathrm {cell}}^{(c)}\big),
\end{equation*}
$$

where

(16) $$
\begin{equation*}
Z=\int \textrm {exp}\big(-\beta H_{\mathrm {cell}}^{(c)}\big) d\mathbf {r}.
\end{equation*}
$$

Thus, the Fokker–Planck equation (Eq. 14) converges to the dynamic equilibrium state and provides a justification for the choice of the corresponding Langevin equation (Eq. 13). There is a single temporal scaling parameter in Eq. 13 and so we can recast it in terms of a nondimensional time $\hat{t}=t|H_{\textrm {s}}|/\gamma$ as

(17) $$
\begin{equation*}
\frac{\partial r_i}{\partial \hat{t}} = - \frac{\partial \hat{H}}{\partial r_i} + \sqrt{\frac{2}{\hat{\beta }\Delta \hat{t}}}\mathcal {N}(0,1),
\end{equation*}
$$

where $\hat{H}=H_{\textrm {cell}}^{(c)}/|H_{\textrm {s}}|$, $\hat{\beta }=\beta |H_{\textrm {s}}|$, and $\mathcal {N}(0,1)$ is a Gaussian distribution of zero mean and unit variance. In writing Eq. 17, we used the fact that the stochastic differential equation (Eq. 13) is solved with a finite time step Δ*t*, where $\Delta \hat{t}=\Delta t |H_{\textrm {s}}|/\gamma$. We thus first present the temporal evolution of the cell morphologies in terms of $\hat{t}$ without explicit knowledge of γ and subsequently estimate γ by comparing with measurements. Details of the numerical procedure to solve Eq. 17 are provided in Supplementary Material Section S4.3.

### Evolution of cell morphology

We first consider the temporal evolution of the cell that is seeded onto the substrate from suspension (a deterministic circular cell morphology of radius 0.96*R*_0_) at time $\hat{t}=0$ with the substrate subjected to cyclic strain (*r* = 0) with ε_amp_ = 0.1 and *f* = 1 Hz. The Langevin equation (Eq. 17) is a stochastic differential equation so that a different solution is generated for every realization of the noise process, i.e. much like in repeated nominally identical experiments a different trajectory of morphological evolution is obtained for every solution of Eq. 17 with the same initial state at $\hat{t}=0$. To generate probability distributions of the temporal evolution of the key observables, viz. the normalized area $\hat{A}$, aspect ratio *A*_*s*_, and cell orientation θ, we simulated 1,000 such trajectories for the cell starting from its state in suspension at $\hat{t}=0$. The probability density distributions are then generated by collecting the 1,000 cell morphologies at each time $\hat{t}$ from the 1,000 Langevin trajectories.

**Fig. 5.**
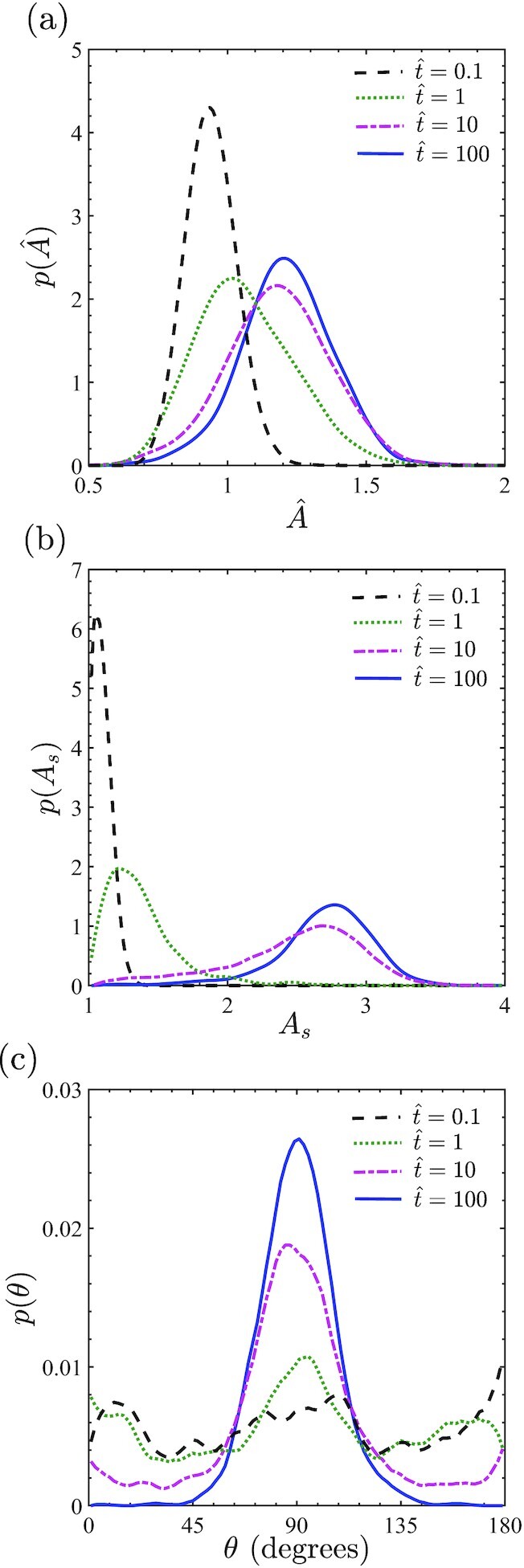
Temporal evolution of the probability density functions of (a) the normalized cell area $p(\hat{A})$, (b) cell aspect ration *p*(*A*_*s*_) and (c) cell orientation *p*(θ) for cells subjected to cyclic strain (*r* = 0) with ε_amp_ = 0.1 and *f* = 1 Hz. Cell are seeded from suspension onto the cyclically strained substrates at normalized time $\hat{t}=0$.

Predictions of the temporal evolution of the probability density functions $p(\hat{A})$, *p*(*A*_*s*_), and *p*(θ) are included in Fig. 5. Since the cell state is deterministic (cell in suspension) at time $\hat{t}=0$ with $\hat{A}=0.92$ and *A*_*s*_ = 1, $p(\hat{A})$ and *p*(*A*_*s*_) are delta functions at $\hat{t}=0$. On the other hand, cell orientation θ is undefined for a circular cell and we assume that *p*(θ) is uniform at $\hat{t}=0$. Soon after the seeding of the cell $(\hat{t}=0.1)$, the distributions $p(\hat{A})$ and *p*(*A*_*s*_) are still highly peaked having diffused out from their initial delta functions. Similarly, at $\hat{t}=0.1$, *p*(θ) has not changed substantially from its initial uniform distribution. With increasing time, the distributions of cell area and aspect ratio become more diffuse with the mean of the distributions shifting to higher values. On the other hand, the cell orientation distribution becomes peaked around θ = 90^○^ implying that cells are starting to orient perpendicular to the cyclic strain direction. In fact, these three distributions nearly converge for $\hat{t}\ge 100$ to the dynamic equilibrium distributions seen in Fig. 3.

### Cells rotate to avoid cyclic strain

Simulations starting from cells in the suspended state are not suited to answer the question: what is the process by which cells avoid cyclic strain? This is because cell orientation at $\hat{t}=0$ is not clearly defined for circular cells. We thus change approach and investigate the cyclic response of cells that have been seeded on the substrate prior to application of cyclic strain and allowed to attain their equilibrium distributions. Cyclic straining of the substrate is then commenced at $\hat{t}=0$ after the static equilibrium has been attained. Not only is such a straining protocol experimentally realizable, it has the advantage that the equilibrated cells are elongated with cell orientation well defined at $\hat{t}=0$.

**Fig. 6.**
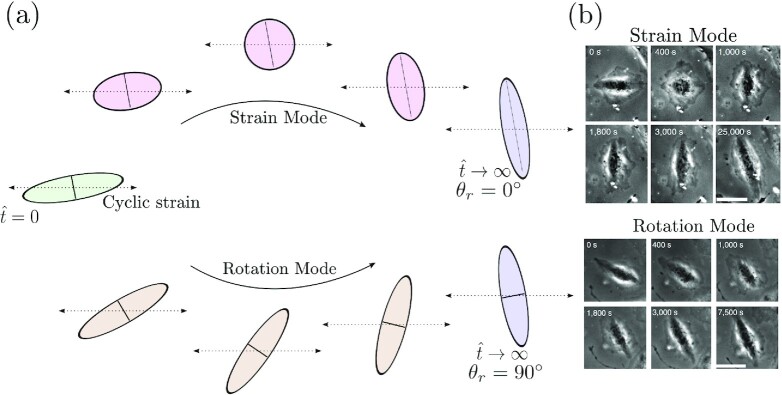
(a) Sketches for the two mechanisms via which the cells could orient away from the cyclic straining direction illustrated here for uniaxial cyclic straining (ε_2_(*t*)/ε_1_(*t*) = 0). In the *strain mode*, cells stretch with negligible cell rotation, while in the *rotation mode*, cells rotate with negligible morphological changes. The sketch shows the reorientation of the cell by 90^○^. To illustrate kinetics of the two processes, we mark a material line (solid black line) corresponding to the minor axis of the initial cell morphology and follow temporal evolution of this material line. In the strain mode, this line does not rotate but stretches to become the major axis, while in the rotation mode, the line rotates by 90^○^ but remains the minor axis. (b) Observations of these two modes in fibroblasts seeded on cyclically loaded substrates. Reproduced from (32).

Our aim here is to differentiate between the two processes by which cells could avoid cyclic strain. These two processes are sketched in Fig. 6(a): (i) the *strain mode*: morphological changes involving cell straining but no cell rotation and (ii) the *rotation mode*: cell rotation with negligible morphological changes. Both these modes have been observed for fibroblasts seeded on cyclically loaded substrates (32); see Fig. 6(b). However, the observations have to-date been unable to quantify the degree of prevalence of the two modes and moreover to the best of our knowledge there exists no model in the literature with the fidelity to differentiate between the two modes of cyclic strain avoidance. To differentiate between these two modes, we will use the following simulation protocol. Recall that all orientations θ of a given morphology of a cell are equally probable in the absence of cyclic strain. We thus consider 50 different cell morphologies specified by couplets of $(\hat{A},A_s)$ selected using the equilibrium probability distributions in the absence of cyclic strain (Fig. 3b and c) and we assign an orientation θ = θ_0_ to all these morphologies. Using these initial conditions, we then run 20 Langevin trajectories on each of the 50 initial cell morphologies (i.e. a total of 1,000 Langevin trajectories for each initial cell orientation θ_0_). By following each of these 1,000 trajectories in time, we can evaluate the cell rotation $\theta _r(\hat{t})$ in each case. We emphasize that $\theta _r(\hat{t})$ is fundamentally different from the cell orientation θ: while θ provides the orientation of the major axis of the ellipse with respect to the $\mathbf {x}_1$-direction, $\theta _r(\hat{t})$ is the rigid body rotation of the cell. Here, we calculate $\theta _r(\hat{t})$ by monitoring the rotation of one of the principal axes of the ellipse in the manner illustrated in Fig. 6(a).

**Fig. 7.**
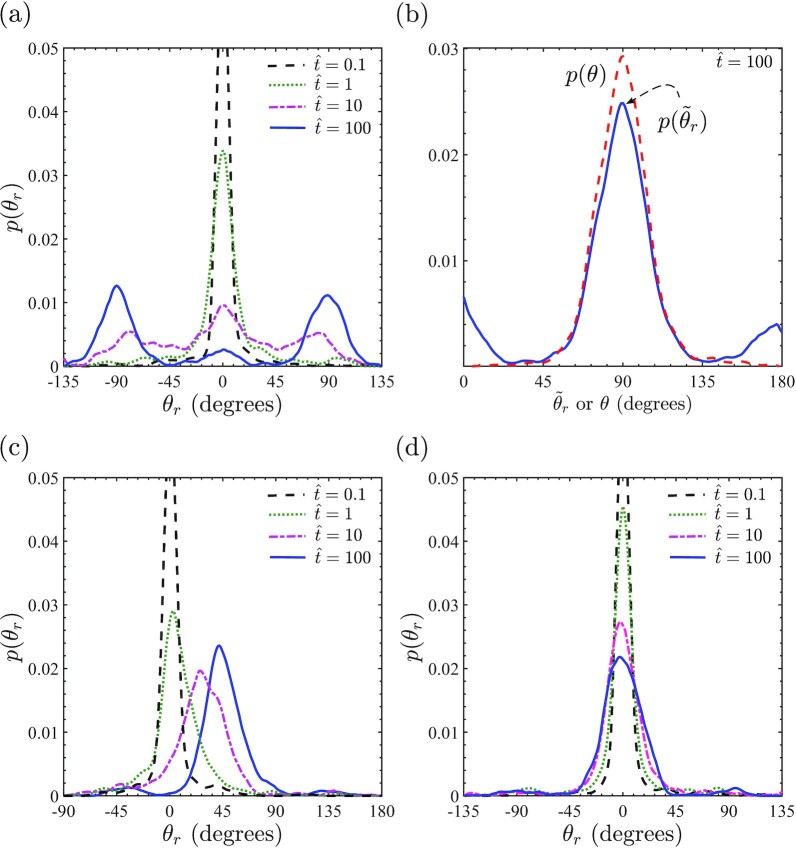
Temporal evolution of the probability density function of cell rotation θ_*r*_ for cells subjected to cyclic strain (*r* = 0) with ε_amp_ = 0.1 and *f* = 1 Hz. Cells are first allowed to equilibrate on the substrate in the absence of cyclic straining with loading commenced at $\hat{t}=0$ for cells initially oriented at θ = θ_0_. Results are shown for (a) θ_0_ = 0^○^, (c) θ_0_ = 45^○^, and (d) θ_0_ = 90^○^. In (b), we show a comparison between the probability density functions of the cell orientation and the auxiliary rotation $\tilde{\theta }_r$ at time $\hat{t}=100$ for the θ_0_ = 0^○^ case. Note that for the *p*(θ_*r*_) distributions, we show a range of θ_*r*_ in each case such that the integral of *p*(θ_*r*_) over the range is at least equal to 0.9. Note that the probability distributions are Dirac delta functions at $\hat{t}=0$, which, in turn, implies that the modes of probability distributions are large at $\hat{t}=0.1$ and hence been cut for clarity.

Evolution of the probability density distributions of *p*(θ_*r*_) for three initial orientations θ_0_ = 0^○^, 45^○^, and 90^○^ with *r* = 0 cyclic straining ε_amp_ = 0.1 and *f* = 1 Hz imposed at $\hat{t}=0$ are shown in Fig. 7(a), (c), and (d), respectively. In all cases, *p*(θ_*r*_) is a Dirac delta function centered at θ_*r*_ = 0^○^ at $\hat{t}=0$. First, consider the θ_0_ = 0^○^ case. With increasing $\hat{t}$, the initial Dirac delta function diffuses out and forms a bimodal distribution with modes at θ_*r*_ = ±90^○^ at time $\hat{t}=100$ when the *p*(θ) distribution is expected to attain its steady state (Fig. 5c). This strongly suggests that the orientation distribution at $\hat{t}=100$ has been attained primarily by the rotation rather than the strain mode with cells rotating by ±90^○^ to align perpendicular to the cyclic straining direction. Recall that while θ varies over the range 0^○^ ≤ θ ≤ 180^○^, θ_*r*_ is unbounded and in fact *p*(θ_*r*_) will continue to evolve even after *p*(θ) has attained a steady-state distribution, although the distribution *p*(θ_*r*_) will maintain a periodicity of ±180^○^ so as to not affect the steady-state distribution of the cell orientation *p*(θ).

To quantitatively verify the claim that the *p*(θ) distribution has primarily been attained by cell rotation, recall that cell rotations in steps of ±π result in the same cell orientation θ. We thus define an auxiliary angle such that $\tilde{\theta }_r=\theta _r\pm n\pi$ and *n* ≥ 0 is an integer that shifts θ_*r*_ so that $0^\circ \le \tilde{\theta }_r\le 180^\circ$. A comparison between the predictions of the probability density functions *p*(θ) and $p(\tilde{\theta }_r)$ at time $\hat{t}=100$ from these Langevin simulations with θ_0_ = 0^○^ are shown in Fig. 7(b). The two distributions are very similar with two exceptions: (i) the peak in $p(\tilde{\theta }_r)$ at $\tilde{\theta }_r=90^\circ$ is smaller than the corresponding peak in *p*(θ), and (ii) two smaller peaks are observed at $\tilde{\theta }_r=0^\circ$ and 180^○^ in $p(\tilde{\theta }_r)$ that are absent in *p*(θ). These differences result from the fact that a small fraction of cells avoid the cyclic straining direction by the strain mechanism without rotating significantly and this results in peaks around $\tilde{\theta }_r=0^\circ$ and 180^○^ and a smaller peak at $\tilde{\theta }_r=90^\circ$. To quantify the fraction of cells that avoid the cyclic strain direction by the strain mode rather than the rotation mechanism, notice that the integral of *p*(θ) ≈ 1 over the range 40^○^ ≤ θ ≤ 140^○^ while over the equivalent range $p(\tilde{\theta }_r)\approx 0.87$, which implies that ${\sim}13\%$ of cells have avoided the cyclic strain direction in this case via the strain mechanism.

A similar conclusion that the rotation mechanism is dominant is obtained from the θ_0_ = 45^○^ case in Fig. 7(c), where the distribution again diffuses out with increasing time but also the mode of the *p*(θ_*r*_) distribution shifts to θ_*r*_ = 45^○^. The *p*(θ_*r*_) distribution is unimodal at the time $\hat{t}=100$ as now the shortest path for cells to align perpendicular to the cyclic straining direction is via a 45^○^ rotation. On the other hand, for the θ_0_ = 90^○^ case (Fig. 7d), the cells are already aligned perpendicular to the cyclic strain direction at $\hat{t}=0$. Thus, with increasing time *p*(θ_*r*_) diffuses out a little (keeping the mode of the distribution fixed at θ_*r*_ = 0^○^) as a few cells rotate toward the cyclic strain direction but this spread in *p*(θ_*r*_) is relatively small.

There are two possible reasons for the rotation mode to be the dominant mechanism of cyclic strain avoidance: (i) *energy-governed*: the energy barrier is lower in the rotational mode, or (ii) *entropy-governed*: the energy barriers are similar for both modes but the number of available phase space trajectories for the rotational mode are more numerous. Rather counter-intuitively, we find that the energy trajectories are very similar for the two modes. This is illustrated in Supplementary Fig. S5a and b, where we include plots of the temporal variations of cell rotation θ_*r*_ and the normalized cell energy $\hat{H}$, respectively, for two specific Langevin trajectories, where the cells were initially oriented at θ_0_ = 0^○^ (these two Langevin trajectories were selected from the 1,000 trajectories used to construct Fig. 7a). The two trajectories correspond to cyclic strain avoidance by the rotation and strain modes as evidenced by the temporal variations of θ_*r*_ (the cell rotation θ_*r*_ ≈ 0^○^ for the strain mode while θ_*r*_ evolves to 90^○^ in the rotation mode). Remarkably, the energy trajectories for the two modes are indistinguishable within the inherent noise of the stochastic Langevin solution (Supplementary Fig. S5b). In fact, the energy barriers along both trajectories are minimal. These findings were confirmed to be consistent across all the Langevin simulations we conducted. We thus conclude that entropy drives the mechanism by which cells avoid cyclic strain: the rotational mode dominates as it has a larger number of low energy barrier trajectories across the phase space by which cells can reduce their free energy and avoid cyclic strain.

### Comparison of predictions with measurements for the temporal evolution of cell orientation

**Fig. 8.**
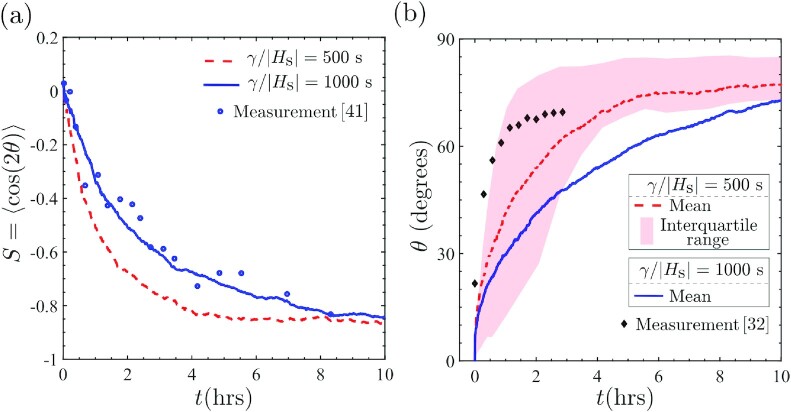
Comparison between measurements and predictions of the temporal evolution of cell orientation with two choices of the damping co-efficient γ/|*H*_s_| = 500 s and 1,000 s. (a) Order parameter *S* = 〈cos(2θ)〉 is compared with measurements of Jungbauer et al. (41). Cells are randomly oriented at *t* = 0 and align perpendicular to the strain direction at large times. (b) Evolution of the orientation of a single cell oriented at θ = 0^○^ and ≈20^○^ at *t* = 0 in the predictions and measurements (32), respectively. The interquartile of the predictions over the 1,000 Langevin trajectories are indicated in (b) for γ/|*H*_s_| = 500 s.

There exists limited data for the temporal evolution of cells seeded on cyclically loaded substrates and here we use data for mouse embryo fibroblasts (32,41) to compare with our predictions and extract the damping co-efficient γ. Jungbauer et al. (41) reported measurements of the orientational order parameter *S* = 〈cos(2θ)〉 with time *t* for uniaxial cyclic loading (*r* = 0) with ε_amp_ = 0.08 and *f* = 2 Hz, with 〈·〉 denoting the ensemble average at a given time *t* over 30 to 50 cells. Our model is ideally suited for comparisons with these measurements as given the statistical nature of the model, the order parameter naturally emerges from the simulations: we interpret 〈·〉 as the ensemble average over our 1,000 Langevin trajectories. Comparisons with our predictions with the choices γ/|*H*_s_| = 500 s and 1,000 s are included in Fig. 8(a). Based on this comparison, γ/|*H*_s_| = 500 to 1,000 s gives good agreement with measurements. Remarkably, Ippolito et al. (8) estimated γ/|*H*_s_| = 600 s by fitting the predictions of the temporal evolution of cell area and aspect ratio with measurements (42) for fibroblasts seeded on substrates without cyclic loading. This suggests that the damping co-efficient is an intrinsic property associated with kinetics of co-operative intracellular processes rather than a parameter that is loading-dependent.

The order parameter measurements of Jungbauer et al. (41) were for cyclic loading of cells, where cells were randomly oriented at *t* = 0. We have demonstrated that the mechanisms of cell reorientation are strongly dependent on the initial orientation of the cells (Fig. 7). Predictions of the temporal evolution of the mean cell orientation, oriented at θ = 0^○^ at *t* = 0, are included in Fig. 8(b) for the same choices γ/|*H*_s_| as in Fig. 8(a) (The predictions in Fig. 8(b) are for uniaxial loading with *f* = 1 Hz and ε_amp_ = 0.1. Symmetry of the loading implies that cells are equally probable to be oriented at θ and π − θ, where θ is an acute angle. For clarity of presentation, we include in Fig. 8(b) only the acute angle θ.). In addition, we also include a band indicating the interquartile range of the 1,000 Langevin trajectories for the γ/|*H*_s_| = 500 s case. We observe that similar to the evolution of *S*, a steady-state cell orientation is attained after ≈10 hours even though now the cells start with an initial deterministic orientation of θ = 0^○^. Lack of data in the literature makes comparisons with measurements more tenuous in this case. However, Livne et al. (32) reported measurements for the temporal evolution of the orientation of a single fibroblast cell initially oriented at θ = 20^○^ with the substrate biaxially strained (*r* = 0.25) with a frequency *f* = 1.2 Hz and strain amplitude ε_amp_ = 0.1. This single measurement is included in Fig. 8(b) and falls within the interquartile range of the predictions with γ/|*H*_s_| = 500 s. This suggests that, in line with predictions, the timescale for cell reorientation is largely independent of the initial cell orientation.

## Concluding remarks

We have developed a cyclic homeostatic ensemble to investigate the distribution of states that cells seeded on cyclically loaded substrates assume. The ensemble is shown to predict a range of experimental observations that include not only the influence of cyclic strain amplitude and frequency on the angular SF distributions but also the influence of cyclic straining on cell morphology. Specifically, the model captures the observed cyclic strain avoidance phenomenon where SFs are aligned primarily perpendicular to the direction of cyclic straining. Moreover, since the model captures the interplay between cell morphology, SF arrangements and cyclic straining, it accurately predicts that cyclic strain avoidance is primarily a consequence of the cells orienting away from the cyclic strain direction rather than a change in the arrangement of SFs within the cells. The more deterministic cell orientation under cyclic loading is also accompanied by a narrower distribution of cell morphologies characterized in terms of cell area and aspect ratio. To the best of our knowledge, no existing model captures such details of the cyclic strain avoidance mechanism and thereby gives physical insight into the physiological importance of cyclic loading on cell behavior.

While the cyclic homeostatic ensemble accurately captures the steady-state cells, assume under cyclic loading, it does not provide insights into the mechanism of cell reorientation. To address this, we constructed a Langevin-type stochastic differential equation  that captures the evolution of cell morphology under cyclic loading so as to finally attain the cyclic homeostatic ensemble. Using the kinetic formulation, we demonstrate that the primary mechanism of cyclic strain avoidance is cell rotation rather than a trajectory involving cell straining. These diverse observations are predicted by the cyclic homeostatic mechanics framework with the strain-rate sensitivity of the tensile stresses generated by SFs, the only mechanosensitive mechanism included in the free-energy model of the cell. Thereby the model provides insights into the key mechanism by which the cell reorients itself.

Taken together, this paper provides a comprehensive computational framework that not only provides mechanistic insights into cyclic cell reorientation but also provides the ability to make accurate quantitative predictions for the distribution of a range of observables, including SF arrangements as well as cell morphologies. The novel insights provided by this statistical mechanics framework can potentially guide tissue engineering strategies, in addition to providing a new understanding of the mechanisms of healthy and pathological cell biomechanical behavior in vivo.

## Supplementary Material

pgac199_Supplemental_File

## Data Availability

All data are available in the manuscript and Supplementary Material.
