# Supplementary material for: Cell reorientation on a cyclically strained substrate
Source: PNAS Nexus. 2022 Sep 22;1(5):pgac199. doi: 10.1093/pnasnexus/pgac199 (PMC9802216; doi:10.1093/pnasnexus/pgac199)
Supplement: pgac199_Supplemental_File [file pgac199_supplemental_file.pdf]

Supplemental material for *Cell reorientation on a cyclically strained substrate*

Shuvrangsu Das,[1] Alberto Ippolito,[1] Patrick McGarry,[2] and Vikram S. Deshpande[1]

[1] *Department of Engineering, Cambridge University, Trumpington St, CB2 1PZ, Cambridge, United Kingdom*

[2] *Department of Mechanical and Biomedical Engineering, National University of Ireland, University Road, H91 CF50, Galway, Ireland*

This PDF file contains supplementary materials, including supplementary text, Figs. S1 to S5, Tables S1 and S2 and supplementary references.

Corresponding author: Vikram S. Deshpande
Email: vsd@eng.cam.ac.uk

Supplementary Material

S1. Morphological ensemble of a cell on a substrate subjected to cyclic strain

We consider an adherent cell on a cyclically strained elastic substrate within a nutrient bath (Fig. 1). We will extend the framework of the homeostatic ensemble [1] to such a dynamic straining situation. The resulting statistical mechanics description is applicable for cells that have attained a dynamic equilibrium under cyclic strain such that key observables including stress-fiber arrangements within cells and cell morphologies are statistically time invariant. Under these circumstances, following Shishvan et al. [1], we will use the ansatz that the distribution of morphologies that the cells assume in this steady or dynamic equilibrium state maximise the morphological entropy of the system.

S1.1. Morphological entropy under cyclic strain

We define the system as the cell and the cyclically loaded elastic substrate to which the cell is adhered. This system is immersed in a nutrient bath so that we can only control macroscopic variables such as the temperature, bath pressure, nutrient concentrations in the nutrient bath and the imposed strain on the substrate. This results in an inherent uncertainty (sometimes referred to as missing information) in microscopic variables (i.e., microstates) of the system such as the cell morphology. This uncertainty stems from the fact that the homeostatic processes within the cell that include (but not restricted to) metabolic processes such as ion pumps, osmosis, diffusion, cytoskeletal reactions, and ATP regeneration are not precisely regulated and this translates to spatio-temporal fluctuations of the cell morphology. The consequent entropy production (or build-up of missing information) is the basis of the framework of homeostatic ensemble.

Shishvan et al. [1] defined a morphological microstate by the mapping/connection of material points on the cell membrane to material points on the substrate, with the remaining cell surface under fixed pressure. In broad terms, the morphological microstate specifies the shape of the cell. In absence of any external loading, as considered by Shishvan et al. [1], the intracellular structure was assumed to be fixed in given a morphological microstate as they assumed a separation of timescales between the evolution of the intracellular structure and the cell morphology. This separation of timescales follows from the fact that the intracellular structure is driven by a range of bio-chemical processes such as actin polymerization, myosin power strokes driving stress-fiber contractility and diffusion of species via unbound cytoskeletal processes within the cell. These processes are relatively fast and limited by the diffusion rate of species such as unbound actin within the cell (chemical reactions and mechanical processes such as wave propagation are typically much faster and thus not the rate limiting processes) [2]. Thus, the timescale to develop intracellular structure, T_{cyto}, is on the order of a few seconds, $T_{\text{cyto}} = \mathcal{O}(1 \text{ sec})$. By contrast, the evolution of cell morphology requires co-operative cytoskeletal processes within the cell such as cytoskeletal reorganization orchestrated by coordinated actin polymerization, dendritic nucleation [3, 4, 5]. The coordinated cytoskeletal processes are much slower and the timescale for the cell morphological evolution T_{cell} is on the order of minutes, i.e., $T_{\text{cell}} = \mathcal{O}(1 \text{ min})$. This enabled Shishvan et al. [1] to assume that the intracellular structure is determined solely by the morphological microstate.

We extend these ideas to cells on substrates subjected to cyclic strain with a period T_{p}. In particular, we will restrict the model to loading where $T_{\text{p}} \ll T_{\text{cell}}$. The physiologically relevant frequency for the cyclic strain is typically around 1 Hz and the two of the three relevant timescales are separated as $T_{\text{cyto}} \sim T_{\text{p}} \ll T_{\text{cell}}$. A consequence of this separation of timescales is that over the period T_{p}, to a very high degree of accuracy, the connections between the cell membrane and substrate remain intact (i.e. the morphological microstate remains unchanged) and the strain-rate of the substrate gets directly transmitted to the portion of the cell membrane adhered to the substrate. Thus, the intracellular structure at a time t within the period T_{p} is determined only by the cell morphology and substrate loading.

We assume there are N_{p} discrete strain states that the substrate and consequently the cell assumes over the period T_{p}. Let $P^{(c)}$ to denote the probability of morphological microstate (c) so that then the probability of the ith state of the system in morphological microstate (c) at time $t = iT_{\text{p}}/N_{\text{p}}$ within period T_{p} is $P^{(i,c)} = P^{(c)}/N_{\text{p}}$. Then, the Gibbs entropy is given by

$$I_T = -\sum_c \sum_{i=0}^{N_{\text{p}}} \frac{P^{(c)}}{N_{\text{p}}} \ln\Big(\frac{P^{(c)}}{N_{\text{p}}}\Big) = \ln N_{\text{p}} - \sum_c P^{(c)} \ln P^{(c)}. \tag{S1}$$

S1.2. The free-energy of morphological microstate (c)

The entropy Eq. (S1) is maximised subject to the constraint that the cell maintains a state of homeostasis. Shishvan et al. [1] demonstrated that for a cell interacting with an elastic substrate that is not subject to cyclic strain, the system on average attains a fixed specific value of the free-energy that is independent of extracellular environment. We will proceed to show in Section S1.3 that while this result does not hold for a cyclically strained of the substrate, there is a closely analogous requirement that specifies the homeostatic constraint of the cell under an imposed cyclic strain. As a first step, we need to define the free-energy of the system for a given morphological microstate state (c) with the substrate subjected to a cyclic strain.

The average Helmholtz free-energy of the system over all cellular fluctuations is given by

$$\langle H_c \rangle = \sum_c \sum_{i=0}^{N_{\text{p}}} \frac{P^{(c)}}{N_{\text{p}}} H^{(i,c)} = \sum_c P^{(c)} \Big(\frac{1}{N_{\text{p}}} \sum_{i=0}^{N_{\text{p}}} H^{(i,c)} \Big), \tag{S2}$$

where $H^{(i,c)}$ is the Helmholtz free-energy of state (i) of the morphological microstate (c). Taking the limit $N_{\text{p}} \to \infty$, Eq. (S2) reduces to

$$\langle H_c \rangle = \sum_c P^{(c)} H^{(c)}, \tag{S3}$$

where

$$H^{(c)} = \frac{1}{T_\mathrm{p}} \int_{t_I}^{t_I+T_\mathrm{p}} H dt, \tag{S4}$$

$H(t) = H^{(i,c)}$ at time $t = iT_\mathrm{p}/N_\mathrm{p}$ and t_I an arbitrary time that defines the initial state over which the averaging performed after the microstate (c) has attained its cyclic steady-state. The entropy maximisation problem can then be expressed as

$$\begin{gathered} \max_{P^{(c)}} I_T = \ln N_\mathrm{p} - \min_{P^{(c)}} \Big(\sum_c P^{(c)} \ln P^{(c)} \Big), \\ \text{subject to constraints} \sum_c P^{(c)} H^{(c)} = H_0 \text{ and } \sum_c P^{(c)} = 1, \end{gathered} \tag{S5}$$

where the ensemble average of $H^{(c)}$ is the homeostatic energy H_0 for an imposed substrate cyclic loading that we will proceed to derive.

S1.3. **The cellular free-energy at dynamic equilibrium**

First consider a free-standing cell (i.e., a cell in suspension and not adhered to the substrate). As discussed by Shishvan et al. [1], suspended cells assume a unique morphological microstate with the Helmholtz free-energy H_s. Also, without loss of generality we can set the Helmholtz free-energy of the isolated elastic substrate to be zero.

Now consider the system in state $(i \in c)$ where the external agency imposes a displacement $u_m^{(i)}$ and corresponding traction $T_m^{(i,c)}$ on the remote surface Γ_e of the substrate with the remainder to the remote surface subjected to zero tractions. To derive the homeostatic constraint, we consider the isolated cell and the isolated substrate (i.e., without the cell adhered to it) subject to the traction $T_m^{(i,c)}$ on Γ_e, i.e., equal to the traction when the cell and the substrate are interacting. Now since the cell is not interacting with the substrate, the corresponding displacement on surface Γ_e is in general different from $u_m^{(i)}$ and we denote the imposed displacement on the isolated substrate by $u_m^{(S|i,c)}$. The corresponding Helmholtz free-energy of the substrate in this state is $H_\mathrm{sub}^{(S|i,c)}$. The cell and substrate are now brought together while keeping the imposed substrate traction fixed at $T_m^{(i,c)}$. Recalling that the temperature of the system remains fixed throughout this process and there are no changes in species in a purely elastic substrate, the Helmholtz free-energy $H^{(i,c)}$ is given by

$$H^{(i,c)} = H_\mathrm{s} + H_\mathrm{sub}^{(S|i,c)} + \int_{\Gamma_e} T_m^{(i,c)} (u_m^{(i)} - u_m^{(S|i,c)}) d\Gamma + \sum_\alpha \chi_\alpha^S \Delta N_\alpha^{(i,c)} \tag{S6}$$

where χ_α^S is the chemical potential of species α in the cell in the suspended state and $\Delta N_\alpha^{(i,c)}$ the change in the number of species α in state $i \in c$ with respect to its number in the suspended state. Denote

$$\Phi^{(i,c)} = H_\mathrm{sub}^{(S|i,c)} + \int_{\Gamma_e} T_m^{(i,c)} (u_m^{(i)} - u_m^{(S|i,c)}) d\Gamma, \tag{S7}$$

and the ensemble average Helmholtz free-energy of the system over all the fluctuations is then given by

$$\begin{aligned} \langle H_c \rangle &= \sum_c P^{(c)} H^{(c)} = \sum_c P^{(c)} \Big(\frac{1}{N_p} \sum_{i=0}^{N_p} H^{(i,c)} \Big), \\ &= H_\mathrm{s} + \sum_c P^{(c)} \Phi^{(c)} + \sum_\alpha \chi_\alpha^S \sum_c P^{(c)} \Big(\sum_{i=0}^{N_p} \frac{1}{N_\mathrm{p}} \Delta N_\alpha^{(i,c)} \Big), \end{aligned} \tag{S8}$$

where

$$\begin{aligned} H^{(c)} &= \sum_{i=0}^{N_\mathrm{p}} \frac{1}{N_\mathrm{p}} H^{(i,c)} = \frac{1}{T_\mathrm{p}} \int_{t_I}^{t_I+T_\mathrm{p}} H dt, \\ \Phi^{(c)} &= \sum_{i=0}^{N_\mathrm{p}} \frac{1}{N_\mathrm{p}} \Phi^{(i,c)} = \frac{1}{T_\mathrm{p}} \int_{t_I}^{t_I+T_\mathrm{p}} \Phi dt, \end{aligned} \tag{S9}$$

with $H(t) = H^{(i,c)}, \Phi(t) = \Phi^{(i,c)}$ at time $t = iT_\mathrm{p}/N_p$ and t_I an arbitrary time that defines the initial state over which the averaging performed when the microstate (c) has attained its cyclic steady-state. Cellular homeostasis dictates that the ensemble average number $\langle N_\alpha \rangle$ is independent of the environment and substrate loading. Thus,

$$\langle \Delta N_\alpha \rangle = \sum_c P^{(c)} \Big(\sum_{i=0}^{N_p} \frac{1}{N_\mathrm{p}} \Delta N_\alpha^{(i,c)} \Big) = 0 \tag{S10}$$

and Eq. (S8) reduces to

$$\langle H_c \rangle = \sum_c P^{(c)} H^{(c)} = H_\mathrm{s} + \sum_c P^{(c)} \Phi^{(c)}. \tag{S11}$$

S1.4. **The equilibrium probability of microstates**

We employ the ansatz that at dynamic equilibrium $P^{(c)}$ attains a distribution $P_{\rm eq}^{(c)}$ that maximises I_T subject to the constraints that $\sum_c P_{\rm eq}^{(c)} = 1$ and $\sum_c P_{\rm eq}^{(c)}(H^{(c)} - \Phi^{(c)}) = H_{\rm s}$. We impose these constraints via Lagrange multipliers $(\Lambda - 1)$ and β, respectively, such that the equilibrium distribution satisfies

$$dI_T = d\Big[\ln N_p - \sum_c P_{\rm eq}^{(c)} \ln P_{\rm eq}^{(c)} - (\Lambda - 1)\Big(\sum_c P_{\rm eq}^{(c)} - 1\Big) - \beta\Big(\sum_c P_{\rm eq}^{(c)} H^{(c)} - H_{\rm s} - \sum_c P_{\rm eq}^{(c)} \Phi^{(c)}\Big)\Big] = 0. \tag{S12}$$

Using the fact that variations $dP_{\rm eq}^{(c)}$ for different microstates are independent and arbitrary while N_p is a constant, we obtain

$$\ln P_{\rm eq}^{(c)} + \beta(H^{(c)} - \Phi^{(c)}) + \Lambda = 0. \tag{S13}$$

The equilibrium distribution then follows as

$$P_{\rm eq}^{(c)} = \frac{\exp\Big[-\beta\left(H^{(c)} - \Phi^{(c)}\right)\Big]}{Z}, \tag{S14}$$

where $Z = \exp(\Lambda) = \sum_c \exp\left[-\beta(H^{(c)} - \Phi^{(c)})\right]$ is the partition function and the Lagrange multiplier β follows from the constraint Eq. (S11).

S1.5. **Special case of "stiff" substrates**

In most cyclic strain experiments, "stiff" elastic substrates are used such that tractions exerted by the cell on the substrate result in negligible substrate deformation. The advantage of such an experimental setup is that the remote strains imposed on the substrate are directly transmitted to the cell allowing for a simple interpretation of the experimental observations. We proceed to reduce the above formulation for this special case.

First consider the free-energy of the system in microstate (c) given by

$$H^{(c)} = \frac{1}{T_{\rm p}} \int_{t_I}^{t_I + T_{\rm p}} H(t)dt = \frac{1}{T_{\rm p}} \int_{t_I}^{t_I + T_{\rm p}} \left[H_{\rm cell}(t) + H_{\rm sub}(t)\right] dt, \tag{S15}$$

where $H_{\rm cell}(t) = H_{\rm cell}^{(i,c)}$ and $H_{\rm sub}(t) = H_{\rm sub}^{(i,c)}$ are the Helmholtz free-energies of the cell and substrate, respectively at time $t = iT_{\rm p}/N_{\rm p}$. For convenience we choose t_I to be a time in the loading history when the imposed cyclic strain is a minimum. Now consider the term $\Phi^{(c)}$. For the assumption of "stiff" substrates where cell causes no additional substrate deformation, $u_m^{(i)} = u_m^{(S|i,c)}$ and $H_{\rm sub}^{(S|i,c)}$ is independent of the microstate (c) and only dependent on the imposed cyclic strain. Thus, from Eqs. (S7) and (S9),

$$\Phi^{(c)} = \frac{1}{T_{\rm p}} \int_{t_I}^{t_I + T_{\rm p}} H_{\rm sub}(t)dt. \tag{S16}$$

The equilibrium probability distribution Eq. (S14) then reduces to

$$P_{\rm eq}^{(c)} = \frac{\exp\Big(-\beta H_{\rm cell}^{(c)}\Big)}{Z_{\rm stiff}}, \tag{S17}$$

where $H_{\rm cell}^{(c)} = (1/T_{\rm p}) \int_{t_I}^{t_I + T_{\rm p}} H_{\rm cell}(t)dt$, $Z_{\rm stiff} = \sum_c \exp(-\beta H_{\rm cell}^{(c)})$ with the corresponding homeostatic constraint

$$\sum_c P^{(c)} H_{\rm cell}^{(c)} = H_{\rm s}. \tag{S18}$$

At equilibrium, the total morphological entropy under cyclic strain is then given by $S_{\rm T} = \ln N_{\rm p} - \sum_c P_{\rm eq}^{(c)} \ln P_{\rm eq}^{(c)}$ such that

$$S_{\rm T} = \ln Q + \beta H_{\rm s}, \tag{S19}$$

where $Q = Z_{\rm stiff} N_p$. Then defining the homeostatic potential for the case of cyclic imposed strain as

$$M = -\frac{1}{\beta}\ln Q = H_{\rm s} - \frac{1}{\beta} S_{\rm T}, \tag{S20}$$

it follows that at equilibrium while the Helmholtz free-energy $H_{\rm cell}^{(c)}$ fluctuates, the homeostatic potential M remains constant.

S2. Cell components and their free-energies

Cells are idealized to comprise a nucleus and cytoplasm. We model the nucleus as a passive hyperelastic material while both active and passive responses of the cytoplasm are modeled. Specifically, we model the active stress-fiber (SF) cytoskeleton and the passive components arising from the elasticity of the membrane, intermediate filaments etc. all again lumped into an hyperelastic model. Thus, the total free-energy is decomposed into the energies of the cytoskeletal and hyperelastic components; see Eq. (8). Under a given cyclic loading, SF components assemble or dissemble via ATP-driven chemical reactions, the kinetics of which depend on cell morphology and imposed substrate loading. The elliptical cell tends to align SFs along its major axis, while the cyclic strain tends to align SFs predominately along the direction perpendicular to the imposed cyclic strain. These two mechanisms could compete or cooperate, depending on whether cell is aligned along the cyclic strain direction or the perpendicular. In the following, we provide a detailed description of the SF kinetics and the associated Helmholtz free-energies of the cell cytoskeleton and passive elements. We present equations for a spatially uniform 2D cell in the $\mathbf{x}_1 - \mathbf{x}_2$ plane with the out-of-plane Cauchy stress $\sigma_{33} = 0$.

S2.1. **Stress-fiber kinetics and the cytoskeletal free-energy $H_{\mathbf{cyto}}(t)$**

The SF kinetics is assumed to be governed by the thermodynamic model proposed by Vigliotti et al. [6] and readers are referred to this original reference for details. Here we provide a brief summary for the sake of completeness and give the relevant equations in the context of a spatially uniform cell as assumed here i.e., the cell is assumed to be a uniformly strained ellipse described by the coefficients (h, k, l) in Eq. (6).

In this model, SFs are characterized by their angular concentrations $\hat{\eta}(\phi)$ and the number of functional units in each stress-fiber $\hat{n}(\phi)$ at an angle ϕ relative to the major axis of the ellipse. The angular concentration $\hat{\eta}(\phi)$ is dictated by formation of new SFs as the fundamental constituents of the SFs transform from their unbound to bound states via ATP-driven reactions. This reaction kinetics is described by a transition state theory at thermodynamic temperature T in terms of the Boltzmann constant k_{B} by

$$\frac{\mathrm{d}\hat{\eta}(\phi)}{\mathrm{d}t} = \frac{\hat{N}_u(1-\hat{\eta}/\hat{\eta}_{\max})}{\pi\hat{n}(\phi)}\omega\exp\Big[-\hat{n}(\phi)\frac{\mu_a-\mu_u}{k_{\mathrm{B}}T}\Big] - \hat{\eta}(\phi)\omega\exp\Big[-\hat{n}(\phi)\frac{\mu_a-\mu_b(\phi)}{k_{\mathrm{B}}T}\Big]. \tag{S21}$$

Here $\hat{N}_u$ is a non-dimensional concentration of the SF units at unbound states which is related to $\hat{\eta}(\phi)$ and $\hat{n}(\phi)$ by conservation of the total number of SF proteins via

$$\hat{N}_u = 1 - \int_{-\pi/2}^{\pi/2} \hat{\eta}(\phi)\hat{n}(\phi)\mathrm{d}\phi. \tag{S22}$$

This SF kinetics is governed by a collision frequency ω combined with the barriers $\mu_a - \mu_u$ and $\mu_a - \mu_b(\phi)$ for the transition of the unbound constituents to their bound state and vice-versa, respectively. In particular, μ_u and $\mu_b(\phi)$ are the enthalpies of a reference number n_R of the unbound constituents units and the bound units at an angle ϕ, respectively, while μ_a is the corresponding enthalpy of the activated state. The enthalpy of the bound units is written in terms of their volume Ω as

$$\mu_b(\phi) = \Psi(\phi) - \sigma_{\mathrm{SF}}(\phi)[1+\tilde{\epsilon}_n(\phi)]\Omega, \tag{S23}$$

where $\Psi(\phi)$ represents the internal energy of the SF at ϕ with n_R units and is given in terms of a non-dimensional constant ν by

$$\Psi(\phi) = \mu_{b0}(1+\nu\tilde{\epsilon}_n^2(\phi)), \tag{S24}$$

with $\tilde{\epsilon}_n$ the nominal strain of a functional unit and μ_{b0} the enthalpy of a SF with n_R units all in their elastic ground state ($\tilde{\epsilon}_n = 0$).

The stress in the stress-fiber $\sigma_{\mathrm{SF}}(\phi)$ is given by a Hill-type constitutive relation [6]

$$\frac{\sigma_{\mathrm{SF}}(\phi)}{\sigma_0(\tilde{\epsilon}_n)} = \frac{1}{2}\Big[\mathrm{erf}\Big(\frac{4\dot{\tilde{\epsilon}}(\phi)}{\dot{\epsilon}_0}+2\Big)+1\Big], \tag{S25}$$

with

$$\begin{aligned}\sigma_0(\tilde{\epsilon}_n) &= \sigma_{\max} & |\tilde{\epsilon}_n| \le \epsilon_p,\\ &= \sigma_{\max}\exp\Big[-\Big(\frac{|\tilde{\epsilon}_n|-\epsilon_p}{\epsilon_s}\Big)^2\Big] & \text{otherwise,}\end{aligned} \tag{S26}$$

where $\sigma_{\max}$ is the isometric tension of the SF and $\dot{\tilde{\epsilon}}(\phi)$ is the true strain-rate in the functional unit. This Hill-type relation includes the strain-rate dependence of the SF stress characterised through the reference strain-rate $\dot{\epsilon}_0$ (Fig. S1a) as well as a strain dependence (Fig. S1b) characterised through two parameters ϵ_p and ϵ_s that govern the regime over which the isometric stress is strain independent and the drop of of the isometric stress as a functional unit is either excessively stretched or compressed.

Now recall that we are considering a 2D cell model with the cell under uniform strain. Moreover, the separation of timescales implies that the morphological microstate of the cell or equivalently the connection between material points on the cell and the substrate remain unchanged over the period when SFs evolve. Thus, the substrate strain-rate is directly transferred to the cell and therefore the SF stretch rate $\dot{\lambda}_{\mathrm{SF}}(\phi)$ for a SF orientated at an angle ϕ with respect to the cell major axis is related to the substrate stretch rates $\dot{\lambda}_1(t), \dot{\lambda}_2(t)$ in the $\mathbf{x}_1$ and $\mathbf{x}_2$ directions, respectively via

$$\dot{\lambda}_{\mathrm{SF}}(\phi) = \dot{\lambda}_1\cos^2(\theta+\phi) + \dot{\lambda}_2\sin^2(\theta+\phi), \tag{S27}$$

where θ is the orientation of the major axis with respect to the $\mathbf{x}_1$ direction. Then integrating this stretch rate from time t_I when the imposed cyclic strain is a minimum and recalling that mean stretch is set by the morphological microstate of the cell gives the

stretch $\lambda_{\mathrm{SF}}(\phi)$ as

$$\lambda_{\mathrm{SF}}(\phi,t)=\bar{\lambda}_{\mathrm{SF}}(\phi)+\Big(\int_{t_I}^{t}\dot{\lambda}_1(t')\mathrm{d}t'\Big)\cos^2(\theta+\phi)+\Big(\int_{t_I}^{t}\dot{\lambda}_2(t')\mathrm{d}t'\Big)\sin^2(\theta+\phi), \tag{S28}$$

where the mean stretch $\bar{\lambda}_{\mathrm{SF}}(\phi)$ is

$$\bar{\lambda}_{\mathrm{SF}}(\phi)=\frac{a_1}{R_0}\cos^2(\phi)+\frac{a_2}{R_0}\sin^2(\phi). \tag{S29}$$

Finally, the nominal strain $\tilde{\epsilon}_n(\phi)$ in the functional unit is kinematically related to the SF stretch $\lambda_{\mathrm{SF}}(\phi)$ through the number of functional units in the SF such that

$$\tilde{\epsilon}_n(\phi)=\frac{\lambda_{\mathrm{SF}}(\phi)}{\hat{n}(\phi)}-1, \tag{S30}$$

and the true stain rate $\dot{\epsilon}(\phi)$ is related to the SF stretch rate by

$$\dot{\epsilon}(\phi)=\frac{\dot{\lambda}_{\mathrm{SF}}(\phi)}{\lambda_{\mathrm{SF}}(\phi)}-\frac{\dot{\hat{n}}(\phi)}{\hat{n}(\phi)}. \tag{S31}$$

The kinetics of the number of functional units $\hat{n}$ driven by the release of the internal energy in functional units as they strain to a more energetically favourable state with this energy consumed in the dissipation associated with the breakage of bonds as functional units and removed/added to the SFs. This kinetics is described by

$$\begin{aligned}\frac{\mathrm{d}\hat{n}}{\mathrm{d}t}&=-\frac{1}{\hat{n}}\left(\frac{\hat{N}_u}{\hat{\Pi}}\right)^2\left[\Psi(\tilde{\epsilon}_n)-\frac{\partial\Psi}{\partial\tilde{\epsilon}_n}(1+\tilde{\epsilon}_n)\right]\frac{\alpha}{\mu_{b0}}\quad\text{for}\quad\frac{\partial\Psi}{\partial\hat{n}}\le 0,\\&=-\frac{\hat{n}}{4}\left[\Psi(\tilde{\epsilon}_n)-\frac{\partial\Psi}{\partial\tilde{\epsilon}_n}(1+\tilde{\epsilon}_n)\right]\frac{\alpha}{\mu_{b0}}\quad\text{otherwise,}\end{aligned} \tag{S32}$$

where α is a rate constant associated with dissipation while $\hat{\Pi}=\int_{-\pi/2}^{\pi/2}\hat{\eta}\mathrm{d}\phi$ is total number of SFs. The reader is referred to [6] for a detailed derivation of the SF kinetics.

The structure of the SFs and the units in the unbound states set the cytoskeletal free-energy such that cytoskeletal Helmholtz free-energy (including contributions from the energy carriers such as ATP; see Ippolito and Deshpande [7]) is given in terms of the chemical potentials χ_u,χ_b of the unbound and bound SF units for an incompressible cell of volume V_{cell} by

$$H_{\mathrm{cyto}}(t)=V_{\mathrm{cell}}(1-f_{\mathrm{nuc}})\rho_0\Big(\hat{N}_u\chi_u+\int_{-\pi/2}^{\pi/2}\hat{\eta}(\phi)\hat{n}(\phi)\chi_b(\phi)\mathrm{d}\phi\Big), \tag{S33}$$

where ρ_0 is the volumetric concentration of SF functional units protein packets within the incompressible cytoplasm and f_{nuc} the volume fraction occupied by the nucleus. The associated chemical potentials are

$$\begin{aligned}\chi_u&=\frac{\mu_u}{n_R}+k_{\mathrm{B}}T\ln(\hat{N}_u),\\ \chi_b&=\frac{\mu_b}{n_R}+\frac{k_{\mathrm{B}}T}{n_R\hat{n}}\ln\left(\frac{\pi\hat{\eta}\hat{n}}{\hat{N}_u(1-\hat{\eta}/\hat{\eta}_{\max})}\right)+k_{\mathrm{B}}T\ln(\hat{N}_u),\end{aligned} \tag{S34}$$

where $\hat{\eta}_{\max}$ is the maximum angular concentration that SFs can attain. For a detailed derivation and discussion of these chemical potentials, the reader is referred to Shishvan et al. [1]. It is emphasized here that we assume that cells are incompressible in three dimensions. In the context of the 2D cells modelled here, the cells are elliptical cylinders and change their area by appropriately adapting their thickness so as to maintain their volume. Moreover, since we are assuming the cells are 2D and spatially uniform there is no variation of the SF organisation within the cytoplasm.

S2.2. The passive free-energy $H_{\mathrm{passive}}(t)$

The total Helmholtz free-energy cell is $H_{\mathrm{cell}}=H_{\mathrm{cyto}}+H_{\mathrm{passive}}$ with the passive energy arising from the elastic deformation of the nucleus and cytoplasm. Thus,

$$H_{\mathrm{passive}}=V_{\mathrm{cell}}\left[f_{\mathrm{nuc}}\Phi_{\mathrm{nuc}}+(1-f_{\mathrm{nuc}})\Phi_{\mathrm{cyto}}\right], \tag{S35}$$

where Φ_{nuc} and Φ_{cyto} are the strain energies per unit volume in the nucleus and cytoplasm, respectively with contributions to elasticity from cell membrane and cell-substrate adhesion complexes lumped into Φ_{cyto}. We assume these strain energy densities are given by an Ogden-type [8] relation in terms of the principal stretches $\tilde{\lambda}_1$ and $\tilde{\lambda}_2$ that the spatially uniform cell is subjected at time t. These stretches are then follow in a manner analogous to Eqs. (S28) and (S29) as

$$\tilde{\lambda}_1(t)=\frac{a_1}{R_0}+\left(\int_{t_I}^{t}\dot{\lambda}_1(t')dt'\right)\cos^2\theta+\left(\int_{t_I}^{t}\dot{\lambda}_2(t')dt'\right)\sin^2\theta \tag{S36}$$

$$\tilde{\lambda}_2(t)=\frac{a_2}{R_0}+\left(\int_{t_I}^{t}\dot{\lambda}_1(t')dt'\right)\sin^2\theta+\left(\int_{t_I}^{t}\dot{\lambda}_2(t')dt'\right)\cos^2\theta, \tag{S37}$$

with the Ogden-type hyperelastic relation is given by

$$\Phi_i=\frac{2\mu_i}{m_i^2}\left[\left(\frac{\tilde{\lambda}_1}{\tilde{\lambda}_2}\right)^{m_i/2}+\left(\frac{\tilde{\lambda}_2}{\tilde{\lambda}_1}\right)^{m_i/2}-2\right]+\frac{k_i}{2}(\tilde{\lambda}_1\tilde{\lambda}_2-1)^2. \tag{S38}$$

This hyperelastic strain energy density function is written in terms of the shear modulus μ_i, area modulus k_i and a strain sensitivity exponent m_i with i representing these parameters in the nucleus or cytoplasm, i.e., $i=\mathrm{nuc},\mathrm{cyto}$.

All the required parameters for the cell free-energy are listed in Tables S1 and S2. The parameters that determines the SF organization as well as the elastic constants for the passive properties of the cytoplasm and nucleus are consistent with Buskermolen et al. [9]. However, Buskermolen et al. [9] did not investigate cyclic loading and hence did not provide the SF kinetic parameters. For SF kinetics, we chose parameters similar to the fast SF remodelling case in [6]. Note that, the normalized free-energy $\hat{H} = H^{(c)}_{\text{cell}}/|H_{\text{s}}|$ is independent of the cell volume V_{cell}, thus all results presented in this study are independent of V_{cell}, and hence this parameter is not specified here. Equally, the results are also independent of the choice of ω and hence this parameter is not listed in Table S1. However, this parameter is used as part of the numerical scheme detailed in Section S4.1 and in all computations presented here we employed $\omega = 20$ Hz.

S2.3. The free-energy of a cell in suspension

The cell in suspension cannot equilibrate itself via reaction forces from the substrate and hence is required to be in a stress-free state. Recall that all microstates are spatially uniform ellipses with principal axes of lengths a_1 and a_2 we use the equilibrium condition that a cell in suspension is stress-free to determine a_1 and a_2 for the cell in suspension. The cell stress comprises contributions from the active contractile forces generated within the stress-fibers and the passive stresses due to elastic deformations. We thus write the total stress Cauchy stress $\boldsymbol{\sigma}$ as

$$\boldsymbol{\sigma} = f_{\text{nuc}}\boldsymbol{\sigma}^{p}_{\text{nuc}} + (1 - f_{\text{nuc}})(\boldsymbol{\sigma}^{a} + \boldsymbol{\sigma}^{p}_{\text{cyto}}), \tag{S39}$$

where $\boldsymbol{\sigma}^a$ and $\boldsymbol{\sigma}^p_i, i = \text{nuc, cyto}$, are the active and passive stresses, respectively, and we have used the fact that the stress-fibers are only present in the cytoplasm. The principal components of $\boldsymbol{\sigma}^p$ are given by

$$\sigma^p_{k,i} = \lambda_k \frac{\partial \Phi_i}{\partial \lambda_k}, \quad k = 1, 2, \quad i = \text{nuc, cyto}, \tag{S40}$$

with the out of plane stress $\sigma^p_{3,i} = 0$ in the 2D context modelled here. The active stress is calculated by averaging the stress of each stress-fiber within a representative volume element [6] and given by

$$\boldsymbol{\sigma}^a = \frac{f_0 \sigma_{\max}}{2} \int_{-\pi/2}^{\pi/2} \hat{\eta}(\phi)\lambda_{\text{SF}}(\phi) \begin{bmatrix} 2\cos^2(\theta+\phi) & \sin(2(\theta+\phi)) \\ \sin(2(\theta+\phi)) & 2\sin^2(\theta+\phi) \end{bmatrix} \text{d}\phi, \tag{S41}$$

where f_0 is the volume fraction of stress-fiber functional units. Solving for $\boldsymbol{\sigma} = 0$ provides a_1 and a_2 and thereby H_{s} that is required to enforce the homeostatic constraint specified by Eq. (5). We note in passing that for a cell that is circular in its elastic resting state, symmetry implies that $a_1 = a_2$ in the suspended state.

S3. Rod-like cells

To analyse the cell orientations for cells seeded on biaxially strained substrates we model cells as rod-like or one-dimensional (1D) cells. The morphological microstate (c) of the cell is then fully described by (λ, θ) where λ is the stretch of the rod and θ its orientation with respect to the $\mathbf{x}_1$-direction of the substrate. The cyclic homeostatic framework remain unchanged except that the ensemble is now over only (λ, θ) and of course we need to specify a model for the cell free-energy.

S3.1. Free-energy of rod-like cells

We reduce the model for 2D cells to the case of rod-like one-dimensional (1D) cells. The model now greatly simplifies as stress-fibers are present in only one direction (i.e., aligned with the 1D cell). Similar to the 2D cells stress-fibers are again characterised by two parameters, $\hat{\eta}$ that gives the concentration of stress-fibers in the 1D stress-fiber bundle and $\hat{n}$ that characterises the number of functional units forming the stress-fibers. The model equations are essentially identical to those for the 2D cell described above. For example, the stress-fiber concentration $\hat{\eta}$ evolves by

$$\frac{\text{d}\hat{\eta}}{\text{d}t} = \frac{\hat{N}_u(1 - \hat{\eta}/\hat{\eta}_{\max})}{\pi\hat{n}} \omega \exp\left[-\hat{n}\frac{\mu_a - \mu_u}{k_{\text{B}}T}\right] - \hat{\eta}\omega\exp\left[-\hat{n}\frac{\mu_a - \mu_b}{k_{\text{B}}T}\right], \tag{S42}$$

with the concentration of unbound functional units now simply given by $\hat{N}_u = 1 - \hat{\eta}\hat{n}$ as stress-fibers are only present in one direction. In fact Eqs. (S23) through (S32) remain unchanged expect that the stress-fiber angle is now synonymous with the orientation θ of the rod-like cell and thus we set $\phi = 0$ and $\bar{\lambda}_{\text{SF}} = \lambda$. Then the cytoskeletal Helmholtz free-energy is given by

$$H_{\text{cyto}}(t) = V_{\text{cell}}(1 - f_{\text{nuc}})\rho_0\left(\hat{N}_u\chi_u + \hat{\eta}\hat{n}\chi_b\right), \tag{S43}$$

where χ_u, χ_b, are given by (S34) while ρ_0 and f_{nuc} are again the concentration of the stress-fiber proteins and the volume fraction of the nucleus, respectively.

The model is completed by specifying the elastic strain energy density functions for the nucleus and cytoplasm as

$$\Phi_i = \frac{k_i}{2}(\tilde{\lambda} - 1)^2, \qquad i = \text{nuc, cyto}, \tag{S44}$$

where

$$\tilde{\lambda}(t) = \lambda + \left(\int_{t_I}^{t} \dot{\lambda}_1(t')\text{d}t'\right)\cos^2\theta + \left(\int_{t_I}^{t} \dot{\lambda}_2(t')\text{d}t'\right)\sin^2\theta. \tag{S45}$$

Then $H_{\text{cell}} = H_{\text{cyto}} + H_{\text{passive}}$ with H_{passive} given by (S35). Finally, to calculate the free-energy of this rod-like cell in the suspended state we first need to estimate λ in the suspended state. Recalling that the suspended cell is stress-free we set the total 1D stress

$\sigma = 0$ where

$$\sigma = (1 - f_{\text{nuc}})\left[f_0 \sigma_{\max} \eta \lambda + k_{\text{cyto}}(\lambda - 1)\right] + f_{\text{nuc}} k_{\text{nuc}}(\lambda - 1). \tag{S46}$$

Solving (S46) gives λ and thereby provides $H_s = H_{\text{cell}}$. The parameters for this rod-like cell are identical to those used for the elliptical cell and listed in Tables S1 and S2.

S3.2. Orientation of rod-like cells under cyclic loading

The analysis for the 2D elliptical cells discussed in the main text can be repeated for the rod-like cells to determine the distribution of the two morphological variables (λ, θ) for substrate loadings specified by (10). These predictions are described in Section 3.3 with predictions included in Figs. S4a and S4b for loading at $f = 1$ Hz and $\varepsilon_{\text{amp}} = 0.1$ and different biaxiality ratios r. In all cases modes of the $p(\theta)$ distribution are observed at $\theta = \tan^{-1}(1/\sqrt{r})$ and $\theta = \pi - \tan^{-1}(1/\sqrt{r})$ corresponding to directions of vanishing substrate strain-rate. This result arises from the fact that for any value of λ in the rod-like cells, H_{cell} is minimised in the direction where the cell (which in this case is synonymous with the stress-fiber bundle) is under isometric conditions such that $\sigma_{\text{SF}} = \sigma_{\max}$. Then via Eq. (4) the probability of observing the cell in this zero strain-rate orientation is a maximum.

S4. Numerical procedures

S4.1. Solution of the stress-fiber kinetics to obtain $H_{\text{cell}}^{(c)}$

Here we briefly describe the procedure to solve the evolution equations for stress-fiber remodeling for a morphological microstate (c) and obtain the corresponding cell energy $H_{\text{cell}}^{(c)}$.

1. There are multiple timescales in Eqs. (S21) and (S32) which makes the corresponding ordinary differential equations (ODEs) stiff. We thus employed the stiff solver *ode15s* in MATLAB to integrate these equations until $\hat{\eta}(\phi, t), \hat{n}(\phi, t)$ achieved a cyclic steady-state.
2. At cyclic steady-state, the temporal variations of $\hat{\eta}(\phi, t), \hat{n}(\phi, t)$ then directly provide $H_{\text{cyto}}(t)$, $H_{\text{passive}}(t)$ and thereby $H_{\text{cell}}(t)$ at every time t over a time period T_p
3. Finally, the cell free-energy $H_{\text{cell}}^{(c)}$ is obtained by averaging $H_{\text{cell}}(t)$ over the time period T_p.

S4.2. Computation of the cyclic steady-state

The Helmholtz free-energy $H_{\text{cell}}^{(c)}$ is computed in the $\mathbf{r} = (h, k, l)$ space for specified values of the substrate strain amplitude ε_{amp} and frequency f. The distribution parameter $\hat{\beta} = \beta|H_{\text{s}}|$ is then computed from the relation

$$\int P_{\text{eq}} \hat{H}(\mathbf{r}) d\mathbf{r} = \frac{H_{\text{s}}}{|H_{\text{s}}|}, \tag{S47}$$

where $\hat{H} = H_{\text{cell}}^{(c)}/|H_{\text{s}}|$ and

$$P_{\text{eq}}(\mathbf{r}) = \frac{\exp(-\hat{\beta}\hat{H})}{\int \exp(-\hat{\beta}\hat{H}) d\mathbf{r}}. \tag{S48}$$

This probability distribution function is then sampled to construct the probability distributions for all observables.

S4.3. Solution of the Langevin-type evolution equation

The numerical procedure to solve the non-dimensional Langevin-type equation (17) is outlined here with $\hat{\beta}$ obtained from the corresponding cyclic steady-state distributions reported in Section 3. The solution provides the temporal evolution of all observables in terms of the non-dimensional time $\hat{t}$ and thus no additional parameters are required to solve Eq. (17). Stochastic differential equations (SDEs) such as the Langevin equation are typically integrated using a Euler forward integration algorithm [10] and we have employed this method to integrate the Langevin SDE as follows:

1. The cell with an prescribed initial configuration is placed on the substrate at time $\hat{t} = 0$.
2. For the microstate at time $\hat{t}$ we evaluate the gradient of the cell free-energy $\partial \hat{H}/\partial r_i$ where $\mathbf{r} = (h, k, l)$ by interpolation of the cell free-energy phase space precomputed in the (h, k, l) space. We emphasize that the free-energy depends on both the substrate cyclic strain amplitude and frequency, which means that the free-energy phase space needs to be computed for every imposed strain frequency and amplitude.
3. For all three degrees of freedom (h, k, l), we then randomly select the value of the noise from the normal Gaussian distribution $\mathcal{N}(0, 1)$ to obtain $\partial r_i/\partial \hat{t}$ from Eq. (17).
4. We then update the current configuration described by (h, k, l) via a forward Euler scheme

$$r_i(\hat{t} + \Delta\hat{t}) = r_i(\hat{t}) + \frac{\partial r_i}{\partial \hat{t}} \Delta\hat{t}. \tag{S49}$$

5. Repeat from steps (ii)-(iv) until $\hat{t} = \hat{T}_{\text{sim}}$ where $\hat{T}_{\text{sim}}$ is the simulation time.

Time evolution was computed by selecting $\Delta\hat{t} = 0.01$ and most trajectories were computed over a period $\hat{T}_{\text{sim}} = 100$. Typically, Langevin simulations were conducted for 50 initial configurations as detailed in the main text and for each of these initial

configurations 20 Langevin trajectories (i.e., different realisations of the noise specified by $\mathcal{N}(0,1)$) were computed. All numerical methods are implemented in in-house codes written in MATLAB. The probability distributions of all observables reported in the main text were then computed from the temporal evolution of the microstate variables (h,k,l).

S5. Effect of the cyclic strain frequency and amplitude

A limited parametric study for the effect of strain amplitude and frequency on key morphological observables is presented here. Specifically, in addition to the reference case of $\varepsilon_{\text{amp}} = 0.1$ and $f = 1$ Hz, we report calculations with $\varepsilon_{\text{amp}} = 0.05$ and $f = 0.5$ Hz.

Figures S2a and S2b show the probability distribution functions (PDFs) for normalized cell area $p(\hat{A})$ and aspect ratio $p(A_s)$, respectively[1]. As expected, the PDFs become wider with either decrease of ε_{amp} or f, and approach to those for the $f = 0$ Hz case. Similar behavior is also observed for the PDF for cell orientations $p(\theta)$ and the stress-fiber angular concentration $\hat{\rho}$, respectively, in Figs. S2c and S2d. Finally, decrease of either frequency or amplitude by half appears to be similar effects consistent with the fact that the cell free-energy depends on contractile strain-rate via the Hill-relation Eq. (S25) for tension generation in the stress-fibers.

[1] Note that probability distribution $p(\hat{A})$ has two peaks $f = 0$ Hz with a significantly larger peak around $\hat{A} = 1$. The smaller peak at lower values of $\hat{A}$ is an artifact of the spatially uniform elliptical shapes assumed here and not been observed when Non-Uniform Rational Basis Splines are used to describe realistic cell shapes [1]. However, given that the focus of this study is on cyclic rather than the static behaviour of cells we do not expect this elliptical approximation to cause significant errors in the predictions

Supplementary Figures

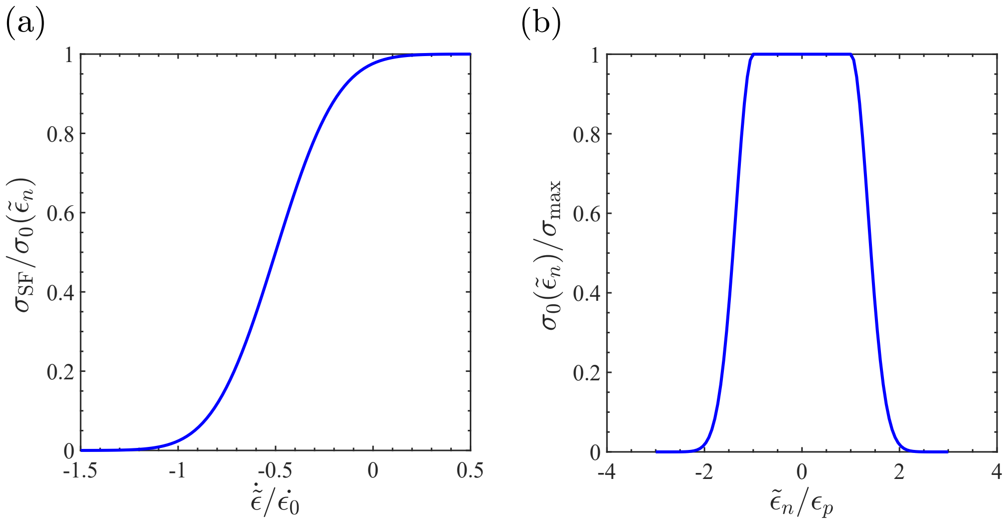

Fig. S1. Sketches showing (a) the stress-fiber stress σ_{SF} normalised by the corresponding isometric value as a function of the normalized strain-rate $\dot{\tilde{\epsilon}}/\dot{\epsilon}_0$ and (b) the isometric stress normalised by the maximum isometric stress value versus the normalised functional unit strain $\tilde{\epsilon}/\epsilon_p$. In (b) the plot is shown for the choice $\epsilon_p/\epsilon_s = 2$.

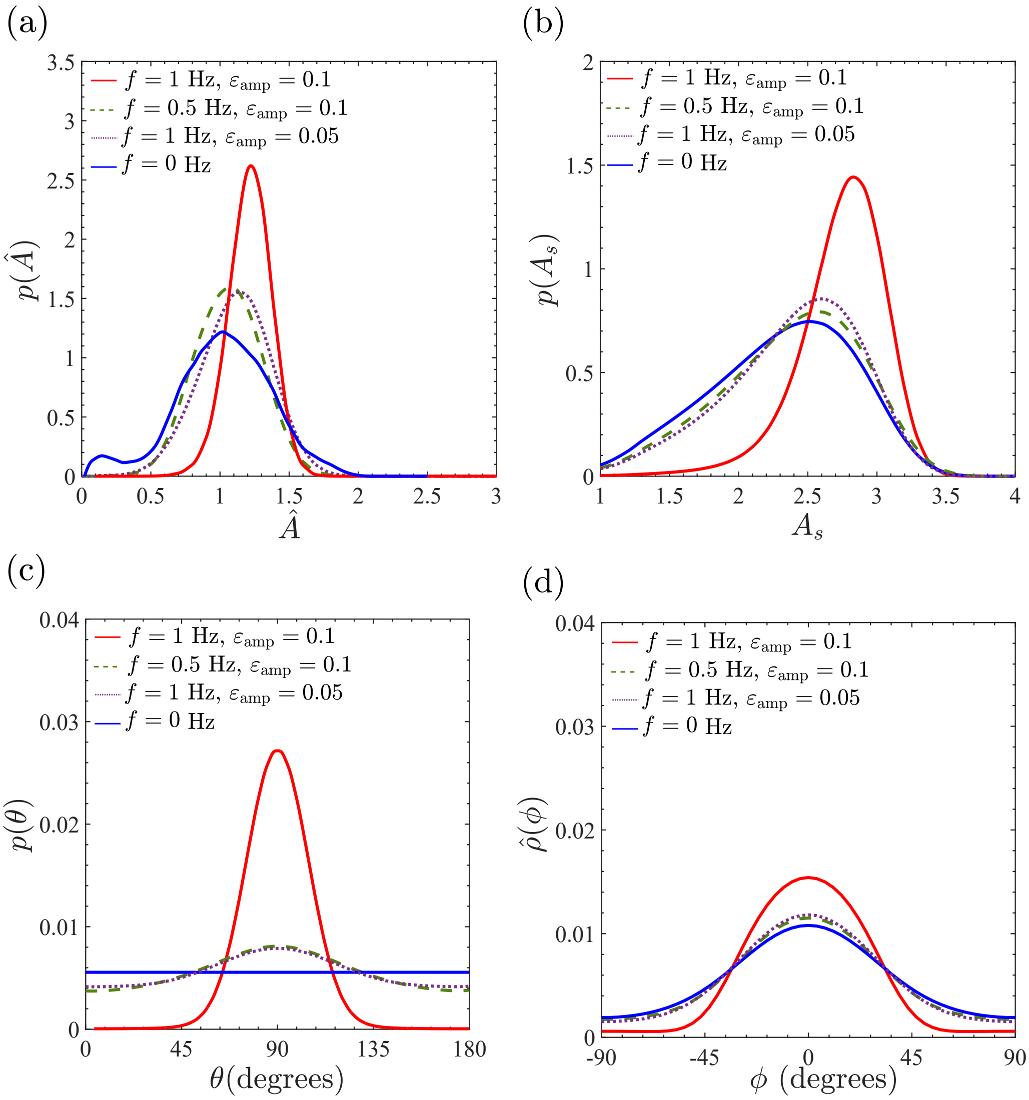

Fig. S2. Effect of cyclic strain amplitude $\varepsilon_{\rm amp}$ and frequency f on key observables for loading with $r = 0$. Probability density functions of (a) the normalised cell area $\hat{A}$, (b) cell aspect ratio A_s and (c) cell orientation θ. (d) Angular distribution of stress-fiber concentration relative to the major axis of the elliptical cell, parameterized by $\hat{\rho}(\phi)$ with the angle ϕ measured with respect to the major axis of the cell. Predictions are show for $\varepsilon_{\rm amp} = 0.1$ with $f = 0.5, 1$ Hz and $\varepsilon_{\rm amp} = 0.05$ with $f = 1$ Hz, together with the case of no cyclic strain $f = 0$ Hz.

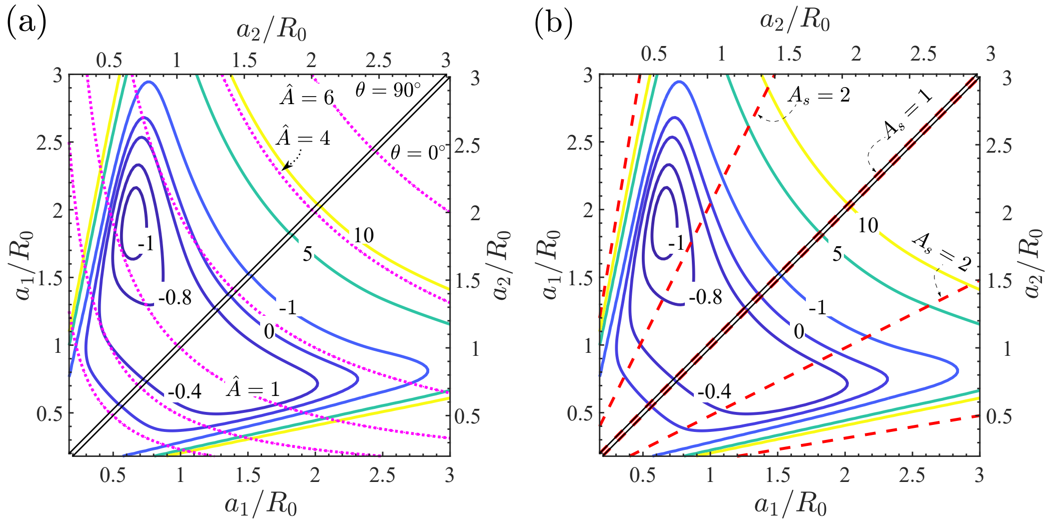

Fig. S3. Replot of the landscape of the normalized energy $\hat{H} = H^{(c)}_{cell}/|H_s|$ in Fig. 4a to included contours of the (a) normalised cell area $\hat{A}$ (dotted lines) and (b) aspect ratio A_s (dashed lines). This plot shows how cell energy changes with cell area and aspect ratio. In brief, $\hat{H}$ varies non-monotonically with both $\hat{A}$ and A_s.

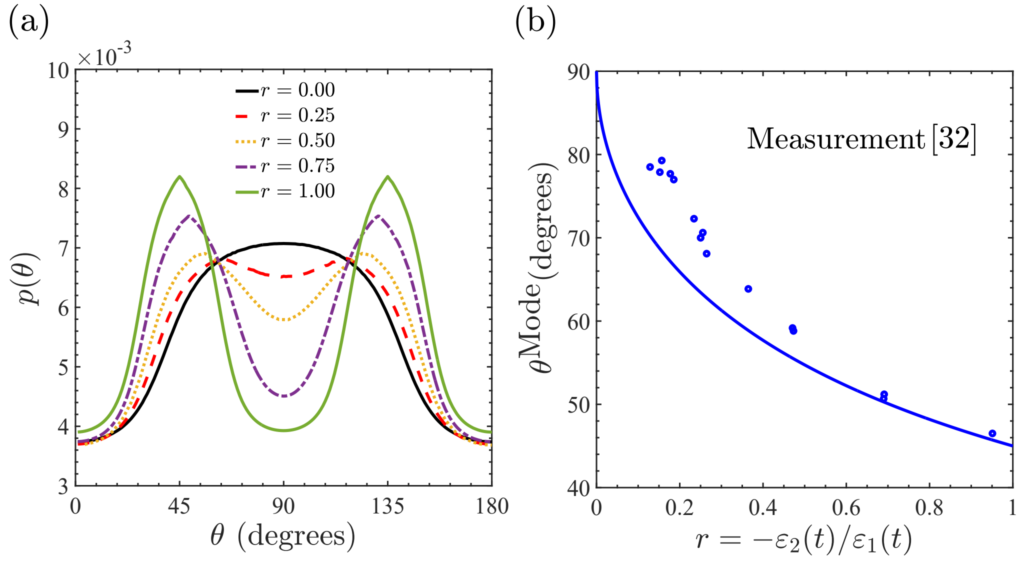

Fig. S4. Predictions of the orientations of rod-like cells under cyclic loading with $f = 1$ Hz and $\varepsilon_{amp} = 0.1$ for different choices of the stretch biaxiality r. (a) Predictions of the probability distribution $p(\theta)$ for selected values of r. For $r > 0$ there are two modes of the distribution corresponding to the directions of vanishing substrate strain-rate. These two directions coincide at $\theta = 90°$ for $r = 0$. (b) Comparisons between predictions and measurements [11] of the mode of the probability distributions (i.e. orientation at which cells are most likely to be observed) as a function of the loading biaxiality r. Note that for $r > 0$ there exist two modes but here for simplicity we just show the mode corresponding to the acute value of θ.

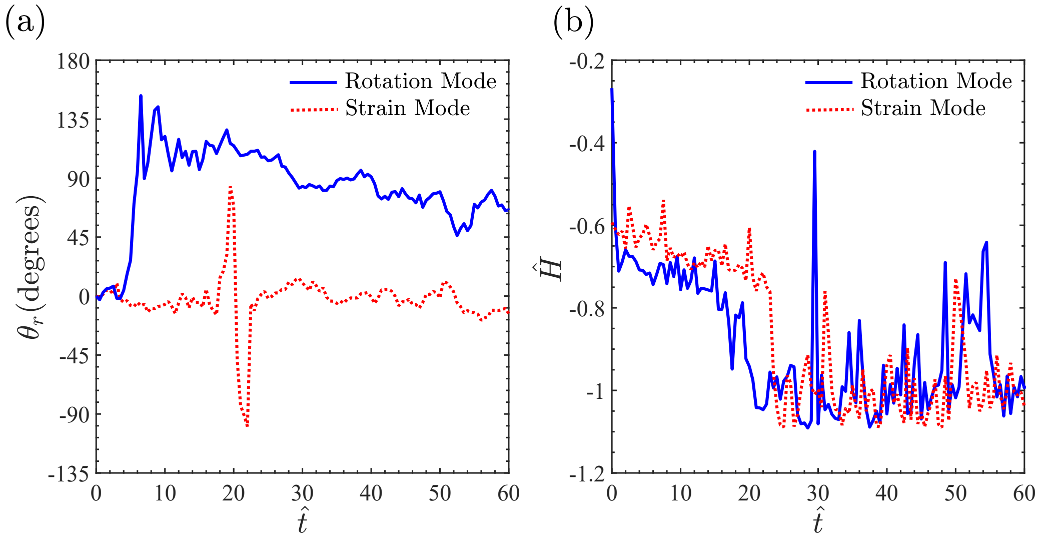

Fig. S5. Two selected temporal Langevin trajectories for cells initially oriented at $\theta_0 = 0°$. The cells were first allowed to equilibrate in the absence of cyclic loading and then subjected to cyclic straining ($r = 0$) with $\varepsilon_{\mathrm{amp}} = 0.1$ and $f = 1$ Hz commencing at normalised time $\hat{t} = 0$. The two selected trajectories correspond to cases with cyclic strain avoidance by the rotation and strain modes. (a) Temporal variation of the cell rotation θ_r and (b) the corresponding variation in the normalised cell-free-energy $\hat{H}$. The spikes in θ_r at $\hat{t} \approx 20$ for the strain mode occur as the cell is approximately circular at that time. In this circular state all orientations of the cell have equal energies and thus are equally likely. As a consequence the Langevin solution samples all orientations. This transient in the cell rotation decays as the cell elongates and there is no residual net rotation from this transient.

Supplementary Tables

Parameters for the SF cytoskeleton	
T	310 K
μ_a	$11k_{\mathrm{B}}T$
μ_u	$8k_{\mathrm{B}}T$
μ_{b0}	$9k_{\mathrm{B}}T$
$\sigma_{\max}$	240 kPa
$\hat{\eta}_{\max}$	1.0
Ω	$10^{-7.101}\mu\mathrm{m}^3$.
ν	1.2
$\dot{\epsilon}_0$	$0.265\ \mathrm{s}^{-1}$
ϵ_p	0.4
ϵ_s	0.2
α	50
n_R	50
ρ_0	$3 \times 10^6\ \mu\mathrm{m}^{-3}$
f_0	0.032
f_{nuc}.	0.2

Table S1. Parameters that characterise the stress-fiber cytoskeleton.

Elastic constants for cytoplasm	
Shear modulus μ_{cyto}	0.3 kPa
Bulk modulus k_{cyto}	50 kPa
m_{cyto}	6
**Elastic constants for nucleus**	
Shear modulus μ_{nuc}	1 kPa
Bulk modulus k_{nuc}	50 kPa
m_{nuc}	15

Table S2. Passive elastic properties of the cytoplasm and nucleus.

References

1. Siamak S Shishvan, Andrea Vigliotti, and Vikram S Deshpande. The homeostatic ensemble for cells. *Biomechanics and modeling in mechanobiology*, 17(6):1631–1662, 2018.
2. James L McGrath, Yanik Tardy, CF Dewey Jr, JJ Meister, and JH Hartwig. Simultaneous measurements of actin filament turnover, filament fraction, and monomer diffusion in endothelial cells. *Biophysical journal*, 75(4):2070–2078, 1998.
3. Aaron Ponti, Matthias Machacek, Stephanie L Gupton, Clare M Waterman-Storer, and Gaudenz Danuser. Two distinct actin networks drive the protrusion of migrating cells. *Science*, 305(5691):1782–1786, 2004.
4. Bruce Alberts, Alexander Johnson, Julian Lewis, David Morgan, Martin Raff, Keith Roberts, and Peter Walter. Molecular biology of the cell. new york: Garland science, 2014.
5. Thomas D Pollard, Laurent Blanchoin, and R Dyche Mullins. Molecular mechanisms controlling actin filament dynamics in nonmuscle cells. *Annual review of biophysics and biomolecular structure*, 29(1):545–576, 2000.
6. A Vigliotti, W Ronan, FPT Baaijens, and VS Deshpande. A thermodynamically motivated model for stress-fiber reorganization. *Biomechanics and modeling in mechanobiology*, 15(4):761–789, 2016.
7. Alberto Ippolito and Vikram S. Deshpande. Contact guidance via heterogeneity of substrate elasticity. *Acta Biomaterialia*, 2021.
8. Raymond William Ogden. Large deformation isotropic elasticity–on the correlation of theory and experiment for incompressible rubberlike solids. *Proceedings of the Royal Society of London. A. Mathematical and Physical Sciences*, 326(1567):565–584, 1972.
9. Antonetta BC Buskermolen, Hamsini Suresh, Siamak S Shishvan, Andrea Vigliotti, Antonio DeSimone, Nicholas A Kurniawan, Carlijn VC Bouten, and Vikram S Deshpande. Entropic forces drive cellular contact guidance. *Biophysical journal*, 116(10):1994–2008, 2019.
10. Grigorii Noikhovich Milstein. *Numerical integration of stochastic differential equations*, volume 313. Springer Science & Business Media, 1994.
11. Ariel Livne, Eran Bouchbinder, and Benjamin Geiger. Cell reorientation under cyclic stretching. *Nature communications*, 5(1):1–8, 2014.
